# Supplementary material for: Physiology of gamma-aminobutyric acid treated Capsicum annuum L. (Sweet pepper) under induced drought stress
Source: PLoS One. 2023 Aug 17;18(8):e0289900. doi: 10.1371/journal.pone.0289900 (PMC10434925; doi:10.1371/journal.pone.0289900)
Supplement: Attachment — Submitted filename: PONE_D_23_13341.pdf [file pone.0289900.s001.pdf]

PONE-D-23-13341.pdf

WORD COUNT 18664

TIME SUBMITTED 06-JUN-2023 06:38AM

PAPER ID 100281754

PLOS ONE

Physiology of gamma-aminobutyric acid treated Capsicum annuum L. (Sweet pepper) under induced drought stress

--Manuscript Draft--

**Manuscript Number:**	PONE-D-23-13341
**Article Type:**	Research Article
**Full Title:**	Physiology of gamma-aminobutyric acid treated Capsicum annuum L. (Sweet pepper) under induced drought stress
**Short Title:**	Physiology of gamma-aminobutyric acid treated Capsicum annuum L. (Sweet pepper) under induced drought stress
**Corresponding Author:**	Babar Iqbal, Mphil<br>Qurtuba University of Sciences and Information Technology - Peshawar Campus<br>Peshawar, Khyber pakhtunkhwa PAKISTAN
**Keywords:**	
**Abstract:**	There is now widespread agreement that global warming is the source of climate variability and is a global danger that poses a significant challenge for the 21st century. Climate crisis has exacerbated water deficit stress and restricts plant growth and output by limiting nutrient absorption and raising osmotic strains in arid, semiarid, and tropical locations across the world, including Pakistan. Worldwide, Sweet pepper is among the most important vegetable crops, and it has been cultivated for thousands of years due to its notorious medicinal and nutritional benefits. Drought is an abiotic stressor that has a negative impact on sweet pepper (Capsicum annuum L.) production. Although γ aminobutyric acid (an endogenous signaling molecule and metabolite) has high physio-molecular activity in plant's cells and is able to encourage tolerance to water scarcity stress, but little is known about its influence on sweet pepper development when applied exogenously. The current study sought to comprehend the effects of foliar γ-aminobutyric acid (GABA) application on vegetative development, as well as physiological and biochemical constituents of Capsicum annuum L. Field experiments were carried out during the 2021 pepper growing season to achieve this goal. GABA (0, 2, and 4mM) concentrations were sprayed on two Capsicum annuum L. genotypes (Scope F1 and Mercury). The results showed that exogenous GABA supplementation (especially 2mM) significantly improved vegetative growth factors like, shoots and roots length, fresh and dry weight, as well as RSR (root shoot ratio), and relative water content (RWC) while decreasing electrolyte leakage (EL). Furthermore, a positive and significant effect on chlorophyll content, carotenoids content, protein content, soluble sugars content, proline accumulation, catalase (CAT), and ascorbate peroxidase (APOX) activity was observed. The application of GABA at 2mM yielded the highest values for these variables. In both genotypes, peroxidase (POD) and superoxide dismutase (SOD) content increased with growing activity of those antioxidant enzymes in treated plants compared to non-treated plants (control). When compared to the other GABA treatments, the 2mM GABA treatment had the highest morphological traits, biochemical composition, and organic compound content. Overall, GABA application can improve development and productivity of Capsicum annuum L. under drought stress circumstances, in addition to cellular mechanisms such as osmoregulation and antioxidant defense.
**Order of Authors:**	Babar Iqbal, Mphil
	Fida Hussain, Phd
	Muhammad Saleem Khan, Phd
	Taimur Iqbal, Mphil
**Additional Information:**	
**Question**	**Response**
**Financial Disclosure**	The author(s) received no specific funding for this work.

Powered by Editorial Manager® and ProduXion Manager® from Aries Systems Corporation

Enter a financial disclosure statement that describes the sources of funding for the work included in this submission. Review the submission guidelines for detailed requirements. View published research articles from *PLOS ONE* for specific examples.

This statement is required for submission and **will appear in the published article** if the submission is accepted. Please make sure it is accurate.

Unfunded studies
Enter: *The author(s) received no specific funding for this work.*

Funded studies
Enter a statement with the following details:

- Initials of the authors who received each award
- Grant numbers awarded to each author
- The full name of each funder
- URL of each funder website
- Did the sponsors or funders play any role in the study design, data collection and analysis, decision to publish, or preparation of the manuscript?
- **NO** - Include this sentence at the end of your statement: *The funders had no role in study design, data collection and analysis, decision to publish, or preparation of the manuscript.*
- **YES** - Specify the role(s) played.

* typeset

Competing Interests

Use the instructions below to enter a competing interest statement for this submission. On behalf of all authors, disclose any competing interests that could be perceived to bias this work—acknowledging all financial support and any other relevant financial or non-financial competing interests.

This statement is required for submission and **will appear in the published article** if the submission is accepted. Please make

The authors have declared that no competing interests exist.

sure it is accurate and that any funding sources listed in your Funding Information later in the submission form are also declared in your Financial Disclosure statement.<br><br>2<br>View published research articles from *PLOS ONE* for specific examples.<br><br>**NO authors have competing interests**<br><br>Enter: *The authors have declared that no competing interests exist.*<br><br>**Authors with competing interests**<br><br>Enter competing interest details beginning with this statement:<br><br>*I have read the journal's policy and the authors of this manuscript have the following competing interests: [insert competing interests here]*<br><br>* typeset	
**Ethics Statement**<br><br>Enter an ethics statement for this submission. This statement is required if the study involved:<br><br>• Human participants<br>• Human specimens or tissue<br>• Vertebrate animals or cephalopods<br>• Vertebrate embryos or tissues<br>• Field research<br><br>Write "N/A" if the submission does not require an ethics statement.<br><br>General guidance is provided below. Consult the submission guidelines for detailed instructions. **Make sure that all information entered here is included in the Methods section of the manuscript.**	N/A

**Format for specific study types**<br><br>**Human Subject Research (involving human participants and/or tissue)**<br>• Give the name of the institutional review board or ethics committee that approved the study<br>• Include the approval number and/or a statement indicating approval of this research<br>• Indicate the form of consent obtained (written/oral) or the reason that consent was not obtained (e.g. the data were analyzed anonymously)<br><br>**Animal Research (involving vertebrate animals, embryos or tissues)**<br>• Provide the name of the Institutional Animal Care and Use Committee (IACUC) or other relevant ethics board that reviewed the study protocol, and indicate whether they approved this research or granted a formal waiver of ethical approval<br>• Include an approval number if one was obtained<br>• If the study involved *non-human primates*, add *additional details* about animal welfare and steps taken to ameliorate suffering<br>• If anesthesia, euthanasia, or any kind of animal sacrifice is part of the study, include briefly which substances and/or methods were applied<br><br>**Field Research**<br><br>Include the following details if this study involves the collection of plant, animal, or other materials from a natural setting:<br>• Field permit number<br>• Name of the institution or relevant body that granted permission	
**Data Availability**<br><br>Authors are required to make all data underlying the findings described fully available, without restriction, and from the time of publication. PLOS allows rare exceptions to address legal and ethical concerns. See the PLOS Data Policy and FAQ for detailed information.	Yes - all data are fully available without restriction

Powered by Editorial Manager® and ProduXion Manager® from Aries Systems Corporation

A Data Availability Statement describing where the data can be found is required at submission. Your answers to this question constitute the Data Availability Statement and **will be published in the article**, if accepted.<br><br>**Important:** Stating 'data available on request from the author' is not sufficient. If your data are only available upon request, select 'No' for the first question and explain your exceptional situation in the text box.<br><br>Do the authors confirm that all data underlying the findings described in their manuscript are fully available without restriction?	
**Describe where the data may be found in full sentences. If you are copying our sample text, replace any instances of XXX with the appropriate details.**<br><br>• If the data are **held or will be held in a public repository**, include URLs, accession numbers or DOIs. If this information will only be available after acceptance, indicate this by ticking the box below. For example: *All XXX files are available from the XXX database (accession number(s) XXX, XXX.).*<br>• If the data are all contained **within the manuscript and/or Supporting Information files**, enter the following: *All relevant data are within the manuscript and its Supporting Information files.*<br>• If neither of these applies but you are able to provide **details of access elsewhere**, with or without limitations, please do so. For example:<br><br>*Data cannot be shared publicly because of [XXX]. Data are available from the XXX Institutional Data Access / Ethics Committee (contact via XXX) for researchers who meet the criteria for access to confidential data.*<br><br>*The data underlying the results presented in the study are available from (include the name of the third party*	All relevant data are within the manuscript and its Supporting Information files.

and contact information or URL).<br>• This text is appropriate if the data are owned by a third party and authors do not have permission to share the data.<br><br>* typeset	
Additional data availability information:	Tick here if your circumstances are not covered by the questions above and you need the journal's help to make your data available.; Tick here if the URLs/accession numbers/DOIs will be available only after acceptance of the manuscript for publication so that we can ensure their inclusion before publication.

Powered by Editorial Manager® and ProduXion Manager® from Aries Systems Corporation

Physiology of gamma-aminobutyric acid treated *Capsicum annuum* L. (Sweet pepper) under induced drought stress

Babar Iqbal[1] , Fida Hussain[1] , Muhammad Saleem Khan[2] , Taimur Iqbal[3]

1 Qurtuba University of Science & Information Technology Peshawar, Peshawar, Pakistan, 1 Department of Botany, Islamia College University, Peshawar, Pakistan, 2 Department of Botany, Islamia College University, Peshawar, Pakistan, 3 Department of Pathology, University of Agriculture, Peshawar, Pakistan

Babariqbal441@gmail.com (BI)

Abstract

There is now widespread agreement that global warming is the source of climate variability and is a global danger that poses a significant challenge for the 21st century. Climate crisis has exacerbated water deficit stress and restricts plant's growth and output by limiting nutrient absorption and raising osmotic strains in arid, semiarid, and tropical locations across the world, including Pakistan. Worldwide, Sweet pepper is among the most important vegetable crops, and it has been cultivated for thousands of years due to its notorious medicinal and nutritional benefits. Drought is an abiotic stressor that has a negative impact on sweet pepper (*Capsicum annuum* L.) production. Although γ aminobutyric acid (an endogenous signaling molecule and metabolite) has high physio-molecular activity in plant's cells and is able to encourage tolerance to water scarcity stress, but little is known about its influence on sweet pepper development when applied exogenously. The current study sought to comprehend the effects of foliar γ-aminobutyric acid (GABA) application on vegetative development, as well as physiological and biochemical constituents of *Capsicum annuum* L. Field experiments were carried out during the 2021 pepper growing season to achieve this goal. GABA (0, 2, and 4mM) concentrations were sprayed on two *Capsicum annuum* L. genotypes (Scope F1 and Mercury). The results showed that exogenous GABA supplementation (especially 2mM) significantly improved vegetative growth factors like, shoots and roots length, fresh and dry weight, as well as RSR (root shoot ratio), and relative water content (RWC) while decreasing electrolyte leakage (EL). Furthermore, a positive and significant effect on chlorophyll content, carotenoids content, protein content, soluble sugars content, proline accumulation, catalase (CAT), and ascorbate peroxidase (APOX) activity was observed. The application of GABA at 2mM yielded the highest values for these variables. In both genotypes, peroxidase (POD) and superoxide dismutase (SOD) content increased with growing activity of those antioxidant enzymes in treated plants compared to non-treated plants (control). When compared to the other GABA treatments, the 2mM GABA treatment had the highest morphological traits, biochemical composition, and organic compound content. Overall, GABA application can improve development and productivity of *Capsicum annuum* L. under drought stress circumstances, in addition to cellular mechanisms such as osmoregulation and antioxidant defense.

Introduction

Climate change has affected the world in many ways, including threatening its plant life (Saqib and Anjum, 2021). Many adverse situations are continuously being faced by plants in both agricultural as well as natural environments. These abrupt changes in climate cause various biotic and abiotic strains and such small variations in climate can prompt the expressions or repressions of several genes in plants, resulting in a decline in crop production (Ayaydin *et al*., 2015; Janeeshma *et al.*, 2016). Climate change isn't only about warming, it also includes increase in the average temperature of the earth, severe droughts, and increase in salinity which leads to further stirring of weather unpredictability and will present even more global challenges in marginal, already stressed agricultural ecosystems. Climate variability also causes Fluctuations in ultraviolet irradiance, evaporative demand and secondary elements like, tropospheric ozone attentiveness, as well as changes in the primary causative agent and carbon dioxide itself (Yeo, 1999).

Many research studies have shown that rise in temperature due to climate variability is likely to affect many agricultural crops such as Triticum aestivum L., Capsicum annuum L., Zea mays and Oryza sativa from 3% to 10% per degree of warming above historic level (Shumaila and Ullah, 2020). Furthermore, most studies suggest that reduce in quality is owing to decline in leaf and grain nitrogen content, protein, macronutrients, and micronutrients such as Mg, Zn, Cu concentration associated with increase of gases such as CO_2, CFCs and O_3, water uptake by soil and creating osmotic stress in plants more mutable in warmer environment (Campbell *et al.*, 2016). To feed the global population, which is predicted to reach up to 9.8 billion by 2050, global agricultural production must be quadrupled. Conversely, natural resources, particularly land and water, are rapidly depleting and eroding. Historical data and model simulations showed a significant danger of drought around the globe (Sofi *et al.*, 2021).

Drought stress is one of the topmost ecological factors that adversely effects the development, production and performance of the plants exclusively, particularly during the vegetative and growth stage by reducing the crop yield produced for economical purposes or existence (Seleiman *et al.*, 2021). Drought stress reduces the rates of photosynthesis and respiration, translocation, ion uptake, and contents of chlorophyll, carbohydrates, nutrient metabolism, and various growth hormones (Iqbal *et al.*, 2020). Water deficiency stress disturbs the equilibrium between ROS (reactive oxygen species) productions and the antioxidant defense system which cause the accretion of reactive oxygen species (ROS) by inducing oxidative stress to protein, lipids membrane and DNA strands disruption (Hasanuzzaman *et al.*, 2020).

The areas around the globe which are half and fully dry are more vulnerable to water deficiency and drought stress. We might see further harshness in the drought stress due to climate change and global warming, mainly during the summer seasons of the year in numerous areas around the world (Munns *et al*., 2011). By the rapid development in the globe inhabitants the forthcoming demands exert extra pressure on natural resources including water and is expected to get further adversative as global warming continues (Gholamin and Khayatnezhad, 2020). The FAO (United Nations Food and Agriculture Organization) states that up to 26% arable land of the total biosphere is distressed by water deficiency stress (Hameed *et al*., 2014).

Pakistan is situated in the Southeast Asia subcontinent and comes in the arid and semiarid zones which directly makes it vulnerable to water scarcity stress problems similar to any other emerging nations in this part of the globe. Pakistan's total region is 881,913 km^2 situated in semi and completely dry regions out of which 0.563 mkm^2 receives a yearly rainfall of less than 60 cm making it hard for crop production. The areas which are mostly rain-fed the total yearly rainfall is considerably less than the crop's water requirement (Anon, 2003). Similarly, the water availability is still not ensured under irrigated conditions throughout the year (Riaz *et al.,* 2013). Pakistan's $1/4^{th}$ (4.9 million hectare) land is prone to drought and the situation is not getting any better (Qamar *et al.,* 2018). All the provinces of Pakistan are facing major water deficient conditions especially the major parts of Balochistan and Sindh since the last few years because the river flow has fallen due to less rainfall. (Chandio, 2012).

Major parts of the country are converting into desserts due to the scarcity of water and low rainfall (Akhtar *et al.,* 2019). Due to low rainfall the reservoirs have also touched to a deceased level and just like any other country, Pakistan is also extracting water out of the ground more than the obligatory rate of restocking 'fossil' ground water at a shocking rate, which can result in a catastrophe (Anon, 2012). This unavailability of water especially for plants is a severe environmental restraint which directly effects the development and photosynthesis process limiting the productivity of plants (Yordanov *et al.,* 2003).

If the water unavailability remains still the same and crops production did not increase for a 212.2 million population, Pakistan may face problems of food crisis in the future. According to an estimated rise in demand by 2025, Pakistan would require 274 million Acre Feet of water supply to suit its demands. While estimations based on research indicates that the availability of water by 2025 is unlikely to vary from the current 191 MAF availability, the difference might widen (Akhtar, 2010). The growing disparity between demand and supply is anticipated to expand further, as per capita water availability in Pakistan fell from 5260 cubic meters in 1951 to 1040 cubic meters in 2010 due to climate change and drought stress, a drop

of more than 400 percent (Sharif *et al.,* 2016). Pakistan breached the water shortage threshold in 2005, and if the current trend continues, the country will reach the extreme water scarcity threshold by 2025 (Ashraf, 2012)

Water deficiency in crop plants causes decreased leaf size, stem elongation, and root proliferation, disturbed stomatal oscillations, cell division as well as expansion and nutrient relations with reduced crop productivity, plant water and its use efficiency problems (Farooq *et al*., 2009). To cope with drought, plants employ a variety of stress-coping mechanisms, including the creation of reactive oxygen species, the generation of stress hormones like ethylene and abscisic acid, and modifications in shoot and root morphology (Chiappero *et al*., 2019; Bhat *et al*., 2021). Drought also rises the degree of abscisic acid (ABA) biosynthesis, which reduces stomatal conductance and thus lessens transpirational injuries (Yamaguchi-Shinozaki and Shinozaki, 2006).

Plants produce different types of organic solutes as common responses to abiotic stresses including osmoprotectant and antioxidant such as proline and soluble sugar (Mohamed and Abdel Hamid, 2013). During drought stress levels of soluble sugars and proline upsurges while reducing the quantities of numerous polysaccharides, starch and total sugars (El Tayeb *et al*., 2006). These compounds guard plants against stresses by cellular modification through the fortification of membranes veracity and enzymes constancy (Farooq *et al*., 2009).

Drought stress affects physiological and biochemical processes such as stomatal opening and closing, which causes lipid and chloroplast oxidation, resulting in changes in pigment and protein formation, CO_2 diffusion, and decreased K+ uptake from plant leaves, which reduces photosynthetic and transpiration rates. (Hassan *et al*., 2019). Plants responds to such reactive oxygen species through initiation of antioxidant defense system present in plants by activating several vital antioxidant enzymes like catalase (CAT), Peroxidase (POD), Superoxide dismutase (SOD), Ascorbate peroxidase (APOX) (Kachout *et al*., 2013). Ascorbate peroxidase enzyme synthesizes ascorbic acid as an electron donor in the antioxidative process of H_2O_2 amelioration, whereas peroxidases work to decompose H_2O_2 via oxidation of co-substrate, which includes phenolic compounds and other antioxidants. Catalase enzyme, on the other hand, converts hydrogen peroxide (H_2O_2) into usable H_2O and O_2 (Yang *et al*., 2019). It has been estimated that abiotic stressors such as salinity stress and drought stress may be responsible for approximately 50% of productivity loss in our chief monetary gain crop plants, whereas food production should increase 38% in 2025 and 57% by 2050 of entire food resources from existing levels globally (Gammoudi *et al*., 2016). To withstand the food supply for constantly growing human population, it is important to yield additional food by worldwide

agriculture (Beddington *et al*., 2012). With the negative effects of abiotic stress, the demand for crop production is increasing day by day, while the number of synthetic fertilizers available is decreasing (Wang *et al*., 2018).

A number of the methodologies to encourage or augment drought tolerance in plants are unjustly costly or laborious. Recently several studies have revealed that PGRs (plant growth regulators) might encourage physiological tolerance in plants to muddle through hostile environmental situations counting drought (Karimi *et al*., 2019). Exogenous administration of plant growth regulators, such as glutamic (Glu), proline (Pro), and polyamines, and γ-aminobutyric acid has recently been identified as an effective approach for alleviating drought damage in plants (Hu *et al*., 2015). GABA is a non-protein amino acid with 4-carbon has the ability to provide multiple protection swiftly to plants against several abiotic stresses under induce tolerance such as stimulation, and mechanical injury, hormonal toxicity, extreme temperatures, salinity stress, waterlogging and water-deficient stress (Jin *et al*., 2019). GABA rapidly accumulates when plants suffer from environmental stresses (Narsai *et al*., 2011). GABA has been demonstrated to have numerous roles in plant species, including control of proline and tricarboxylic acid (TCA) cycle metabolism, maintenance of carbon, oxidative damage mitigation and nitrogen metabolism linked with tolerance to abiotic stressors (Shelp *et al*., 2012; Signorelli *et al*., 2015). In the regulation of plants growth and tolerance against various abiotic stresses GABA is numerously involved in countless physiological and biochemical functions such as C–N fux for tricarboxylic acid cycle, carbon/nitrogen metabolism regulation of redox status and osmotic pressure, (Salah *et al*., 2019). All these reports against a number of abiotic stresses in plant species treated with GABA suggests that there is potential means of enhancing tolerance in plants.

Capsicum annuum L. belonging to family Solanaceae has a lot of medical as well as economic importance and is cultivated all over the world (Guo *et al*., 2016). It is considered the 3rd greatest monetary gain crop of family Solanaceae right after potato and tomato (Shumaila and Ullah, 2020). This plant is indigenous to South and North America. Mexico is a major pepper producer, and it also grows pepper seeds in the mid-hills during the off-season (Kumar *et al*., 2006). Chilies are grown on approximately 62,000 hectares in Pakistan, with a yearly production of approximately 131,000 tonnes (Naz *et al*., 2006). Sindh and Punjab provinces are key lands for the production of sweet pepper, accounting for 96 percent of chilli cultivation in Pakistan (Kumar *et al*., 2018).

According to the Govt. of Pakistan, (2005-2006) Pakistan's overall pepper output ranges from 120.46 thousand tons from 59.4 thousand hectares of total cultivated land to an

average output of 2.028 tons per hectare (Khan *et al*., 2009). The difference in production quantity between Pakistan and other pepper producing countries of the world is greater because pepper production in our country is primarily influenced by abiotic stress factors such as salinity, drought, and high temperatures, particularly in Sindh, Pakistan's main pepper producing province (Nadeem *et al*., 2014). These farming areas have shrunk by 2.68 percent from 5.8 million hectares as a result of abiotic and biotic components of the environment (Bibi, 2016).

Sweet pepper is considered as the cash crop and some of the supreme valuable crops in many countries including Pakistan where its share is 1.5% in the GDP and is the sixth highest sweet pepper exporter all over the world (Khan *et al*., 2009). Sweet pepper is extensively cultivated in Pakistan on 57.4 thousand hectares producing 188.9 thousand tons with a regular yield of 3.3 tons/hec for local usage and export throughout the year 2009–2010 (Pakistan Economic Survey 2010–2011).

Capicum annuum L. is mostly grown in rain fed regions throughout the world and mostly undergo water deficient conditions in the summer season of the year due to less rainfall caused by climate change (Thomas *et al*., 2017). Those areas which are already water deficient the total production of sweet pepper is grimly affected by drought which at the same time may bring changes in the plant's characteristics such as physio-biochemical, morphological, and metabolic modification in many plant's organs (Campos *et al*., 2014).

The goal of the current research was to evaluate the biochemical, physiological, and growth of *Capsicum annuum* L. (Scope F1 and Mercury) under various exogenous GABA application levels, as well as to identify its potential efficacy in removing the negative effects of water deficiency stress and to learn how adaptable GABA is in controlling crucial metabolic processes by improving drought tolerance in *Capsicum annuum* L. exposed to various intensities of drought.

2. Materials and methods

2.1. Site description and experimental design

Field trials were carried out in natural environment at the Department of Botany, Qurtuba University Peshawar, (33° 58' 49.0" N and 71° 26' 45.9" E) during the pepper growing season of 2021. Peshawar has a tropical climate. Temperature ranges from 5° degrees Celsius (in January) to 39° degrees Celsius (in June). Prior to planting, soil sample (0 to 30 cm) was taken from the experimental location for soil physio-chemical investigation. The soil's chemical properties were as follows: pH = 6.2, EC = 0.383 ds/m (McLean, 1983), % field capacity 8.69–31.02, organic carbon (C) content was 0.55 % (Nelson and Sommers, 1982), nitrogen level was 0.027 g/kg (Keeney and Nelson, 1982), and accessible phosphorus level was 0.053 g/kg (Jackson, 1973) and available potassium was (K = 0.014 g/ kg) (Hanway and Heidel, 1952). The soil moisture content was maintained at 43 %. The soil textural class was silt loamy as calculated by the hydrometer method (Bouyoucos, 1962).

The seeds of two *Capsicum annuum* L. varieties, Mercury and Scope F1 were supplied by Pakistan's National Agriculture Research Center (NARC). This is a high productive crop with an average weight of 50–60 g four lobed fruit that is dark green in color. Harvesting period was 70–75 days after transplanting. The seeds were planted in clay containers (height 20 cm, lower and upper diameter 18cm, while thickness is 2cm), which were filled with 2kg of soil in a 3:1 ratio (sand, loam) with well-composted farmyard manure. Pots were kept at a nursery with an area of 3 × 1.5 m^2 and placed in a complete randomized block design (RCD) 5 cm apart from each other to allow for proper air movement and were protected from rain. For optimum development, the seedlings were cultivated in pots that were exposed to sunlight.

Watering was done on a regular basis to keep the soil wet, and weeds were removed by hand. For each treatment, three replications were carried out. From seed cultivation through sampling, the study took 4 months. The *Capsicum annuum* L. seeds were sterilized in one percent sodium hypochlorite (NaOCl) for ten minutes before being rinsed in distil water for one minute. The normal temperature and relative humidity throughout plant's growth were 16/38 °C (day/night) and 60–70%, correspondingly. A modest quantity of polyoxyethylenesorbitan monolaurate (Tween-20) was added as a surfactant to a GABA solution to improve leaf adherence. After 15 days of germination the plants were divided/thinned into three per replication. The GABA treatment began at the fourth leaf stage after the water deficit treatments were properly established.

GABA concentrations of 0, 2, and 4mM were employed. The GABA was sprayed onto both sides of the leaves in such a way that it thoroughly gets soaked. The exogenous application of foliar spray was done two times. The first foliar application of GABA occurred three days beforehand of water deficiency stress. Two weeks after the drought stress was recognized, the second spray was administered. As a result, all the plants were subjected to 3 stages of water scarcity stress from 72 hours afterward the foliar application until trials (which lasted about four months): stressed free circumstances (the control group i.e., complete irrigation), moderate stress (50 % of field capacity), and severe stress (30 % of field capacity).

Irrigation was complete throughout the early phases of plant growth. These circumstances were preserved throughout the experiment. 54 sweet pepper plants were collected at eighty percent maturity in this experiment (in the green stage). Three duplicates of leaf samples were used. Because each replication was taken from three pots, there were a total of nine plants in each treatment group of both kinds. The physiological parameters were evaluated in the top portion of the seedlings (leaf), and the samples were rapidly iced up in liquid nitrogen after harvest and stored at -80°C for examination of growth components, osmoprotectants, and plant antioxidant enzymes, as well as germination parameters. Throughout the trial, all normal practices for healthy and disease and pest-free crops were followed on a regular basis.

2.2. Chemical investigation of rhizospheric soil

2.2.1. Estimation of soil's electrical conductivity (EC) and pH

A suspension of 1:1 (soil: water) was used to evaluate the pH of rhizospheric soil. The 10-gram samples of the soil were combined with distilled water of 10 ml and agitation of the mixture was done for one hour to make a homogenous mixture. For the filtration of suspension filter paper (Whatman No. 42) was used. The EC and pH of the suspension was determined (McLean, 1983).

2.2.2. Estimation of Field capacity percentage

Ullah *et al*. (2016) approach was employed to compute the field capacity % of rhizospheric soil.

$$\%\, field\ capacity = \frac{Weight\ of\ wet\ soil\ (g) - Weight\ of\ dry\ soil\ (g) \times 100}{Weight\ of\ dry\ soil\ (g)} \qquad \text{(Eq 1)}$$

2.2.3. Estimation of Macronutrient

In a flask 0.5-gram of air dehydrated and sieved soil sample was placed, and 20 ml of extracting solution was added to it (0.05N HCl + 0.025N H_2SO_4). It was filtered using Whatman No. 42 filter paper after being agitated for 15 minutes on a mechanical shaker. The

extracting mixture was diluted to a volume of 50 ml in a conical flask. The atomic absorption method was used to determine the elements K+, C, N, and P (Hussain *et al.*, 2011).

2.2.4. Estimation of Moisture content percentage (MC %)

To evaluate the soil's moisture content, a sample was obtained from the containers at a depth of five inches. The dirt was dehydrated for 72 hours at 70 °C in an oven beforehand being weighed. The soil's moisture content was determined using the formula below. (Ullah *et al.*, 2016).

$$Moisture\ content\ \% \frac{Fresh\ weight\ of\ sample - dry\ weight\ of\ sample \times 100}{Fresh\ weight\ of\ sample} \quad \text{(Eq 2)}$$

2.3. Agronomic characteristics

2.3.1. Growth parameters

A digital balance was used to measure the fresh weight of pepper seedling shoots in each replication immediately after harvest. After oven drying at 70 °C until stable mass was obtained, the dry weight of the shoots was recorded (Ahmed *et al.*, 2014). A meter rod was used to measure the length of the sweet pepper roots and shoots. The roots of the sweet pepper plants were gently taken from the containers, and the fresh weights of the roots was measured using a digital balance after numerous washings with water. They were then roasted at 70 °C to ascertain the dry weights of the roots of sweet pepper plant. (Ahmed *et al.*, 2014).

2.3.2. Root shoot ratio (RSR)

Chuyong and Acidri's (2017) method was used to determine this parameter. The dry mass (g) of both the root and shoot was measured after being keep in an oven for 72 hours for further calculations.

$$RSR = \frac{rootdrymass}{shootdrymass} \quad \text{(Eq 3)}$$

2.3.3. Relative water content

To determine the relative water content (RWC) of leaves a technique reported by Ali and Ashraf (2011) was employed. During this process, completely grown leaves were chosen and their fresh weights was noted. These leaves were then put in test tubes having distil water at room temperature for 3 hours approximately. Their turgid weights were weighed. The dry weight of the leaves was determined after drying them for 48 hours at 70 °C. The leaf relative water content was obtained using the equation below:

$$RWC\ (\%) = FW - \frac{DW}{TW} - DW\ \times 100 \quad \text{(Eq 4)}$$

2.3.4. Electrolyte leakage

The electrolyte leakage of *Capsicum annuum* L. leaves were estimated conferring to method of Barranco *et al*. (2005). Garden-fresh leaves were sliced into 0.1-g discs. To remove contaminants and residues from the sample's, distilled water was administered. After that, the leaf samples were put in 10-ml test tubes filled with distilled water. Before measuring solution's primary electric conductivity, the tubes were incubated for 30 minutes at 30 °C (EC1). While measuring secondary electric conductivity (EC2), the tubes were submerged in a 100 °C water bath for 15 minutes. Finally, the overall EC was computed by the following equation:

$$EL\ (\%) = \left(\frac{EC1}{EC2}\right) \times 100 \qquad \text{(Eq 5)}$$

2.4. Investigation of plant's Physiological and biochemical components

The physiological and biochemical process detailed methods are given below.

2.4.1 Estimation of Total chlorophyll content (TCC)

The leaves chlorophyll content was determined using the Zou *et al*. (2017) method. The pepper plant's fresh leaves of 0.5-gram were chopped up in 80 % acetone with a pestle and mortar and after 24 hours of incubation in the dark it was centrifuged. The aforementioned was centrifuged at 2000 rpm for approximately 5 minutes to pure the suspension. Chlorophyll content was then determined using supernatant. A spectrophotometer was used to measure absorbance at 649 nm, 663 nm and 470 nm for chlorophyll "a", chlorophyll "b" and carotenoids against an 80 % acetone blank.

2.4.2. Estimation of Soluble sugar content (SSC)

For the determination of soluble sugar content, the usual Marcinska *et al*. (2017) methodology, with minor changes was employed. The pepper plant's 0.5-gram foliar material was crushed and then homogenized in 10ml distilled water before adding 1 ml of 30% phenol to 0.1 ml supernatant and incubating the trials for 20 minutes. After incubation, 5.0 ml of rigorous sulfuric acid was supplemented, and values at 420 nm were taken.

2.4.3. Estimation of Total proline content (TPC)

For the approximation of proline content, Plazek *et al*. (2013) method was used. By homogenizing fresh leaves (0.5-gram) in 10 ml of 3% (w/v) sulfosalicylic acid and then filtering the suspension. Combine 2 ml filtrate, 2 ml glacial acetic acid, and 2 ml acid ninhydrin (1.87 g ninhydrin + 40 ml glacial acetic acid + 30 ml of 6 M phosphoric acid) in a test tube and

warmed it at 100°C for 1 hour. The entire process was halted in an ice bath. Toluene of 4ml was used for extraction, and readings at 520 nm was measured.

2.4.4. Estimation of soluble protein content (SPC)

The SPC of the leaf was computed by the methodology of Lowery (1951) via BSA as standard.

a. 1 gram of monosodium phosphate (MSP) was liquefied in distil water.
b. 9.11 gram disodium phosphate (DSP) liquefied in 170 ml of distil water.

To achieve the desired pH of 7.5 for the phosphate buffer, monosodium phosphate of 32ml and disodium phosphate of 168ml were mixed. Reagent A was prepared by dissolving 3 gram of sodium carbonate (Na_2CO_3), 1.5 gram of Na-k tartrate and 0.6 gram of Sodium hydroxide (0.1 N) in 150 ml of distil water. For preparation of Reagent B $CuSO_4.5H_2O$ (0.125 gram) was dissolved in 25ml of distil water. Reagent C was created by combining solution a (150ml) and solution b (3ml). Reagent D: 10 ml of foline phenol reagent was diluted with 10 ml of distil water in a 1:1 ratio.

In phosphate buffer solution of 1ml of pH 7.0, foliar material of 0.5-gram were grounded and homogenized for 10 minutes. A total volume of 1 ml was obtained by adding distil water to 0.1 ml of extract and mixing it with 1 ml of reagent containing [0.75 g sodium carbonate (Na_2CO_3), 0.1 N sodium hydroxide (NaOH), and 0.37 g sodium potassium tartrate (Na-K tartrate) in distilled water of 40ml]. Shake the solution for ten minutes before adding 0.1 ml foline phenol reagent to a test tube and incubating for thirty minutes. At 650 nm, the absorbance of the sample was noted using a spectrophotometer.

2.4.5. Estimation of peroxidase activity (POD)

The methodology of Asthir *et al*. (2009) was employed for peroxidase activity. The plant's fresh leaves of 0.5-gram were homogenized and centrifuged for 20 minutes with a 2 ml solution containing [0.2 ml phosphate buffer solution (pH 7.0), 12.5 g (PVP) and 4.6 g ethylene diamine tetra acetic acid (EDTA) in distilled water of 125ml]. The reaction mixture (3 ml) included 0.1 ml supernatant, 1.3 ml methyl ethyl sulfonic acid (MES) buffer (970 mg MES in 50 ml distil water), 0.1 ml phenyl diamine (36 mg in 4 ml distil water), and a droplet of 0.3 % H_2O_2. A spectrophotometer was used to record the changes in absorbance for 3 minutes at 485 nm.

2.4.6. Estimation of superoxide dismutase activity (SOD)

The typical approach of Yuan *et al*. (2011) was used for the measurement of SOD concentration in the leaves. The reaction mixture of 3 ml containing supernatant of 0.1 ml, 0.72 ml methionine (58 mg methionine dissolved in 30ml distil water), nitroblue tetrazolium (NBT) of 0.72 ml (1.89 mg nitroblue tetrazolium dissolved in 30ml distil water), 0.72 ml Ethylenediaminetetraacetic acid (1.1 mg Ethylenediaminetetraacetic acid dissolved in 30ml distil water) and riboflavin of 0.72 ml (0.02 mg riboflavin dissolved in 30ml distilled water) after that the mixture was incubated for approximately a period of 30 minutes in the dark as well as in the light and spectrophotometer was used for taking readings at 560 nm.

2.4.7. Estimation of catalase activity (CAT)

The recommended technique of Tybursk *et al*. (2009) with some modification was used to determine Catalase content (CAT) in foliar material of *Capsicum annuum* L plant. The reaction mixture (1 ml) was made up of 0.4 ml of 100mM PBS (pH 7.0), 0.4 ml of 30 % H_2O_2, and enzyme extract of 0.2 ml. The decrease in absorbance value at 240 nm after 3 minutes of H_2O_2 breakdown was measured.

2.4.8. Estimation of ascorbate peroxidase activity (APOX)

Content of APOX was measured by the recommended protocol of Salimi *et al*. (2016). The reaction mixture of 1ml was made by homogenizing fresh foliar material (0.5g) with 5ml PBS solution and centrifuging it at least for ten minutes. Enzyme extract of 0.1 ml was combined with 1.8ml of 50mM phosphate buffer with a pH of 7.0, and 0.1ml of 0.5mM ascorbic acid, and 1mL of 30 % H_2O_2. The absorbance was measured as a reduction occurred at 290 nm for three minutes when compared to a blank 30 % H_2O_2.

2.4.9. Statistical analysis

A factorial design with varied levels of induced drought stress was used for the statistical analysis. Statistix 10 and SPSS Statistics 25 were used to calculate data for several germination parameters, physiological and biochemical components (SPSS Inc, Chicago IL). Two-way anova, principal component analysis, regression and correlation, and the Least Significant Differences (LSD) test were all performed using this programme. For bar graphs, Excel software was also utilized. Different P values were used to determine significance, as well as mean separation and standard deviation (Shumaila and Ullah, 2020).

3. RESULTS

The growth parameter, physiological and biochemical components under induced drought stress of *Capsicum annuum* L. in response to several GABA treatments were investigated.

3.1. Effect of GABA on roots length, roots fresh weight, roots dry weight, relative water content and electrolyte leakage under induced drought stress.

Results of table 2a and 2b indicated the use of GABA foliar spray resulted in maximum values of RL (roots length), RFW (roots fresh weight), RDW (roots dry weight), electrolyte leakage (EL), and Relative water content (RWC) during 50% and 30% field capacity drought stress in both genotypes, Scope F1 and Mercury. The use of 2mM GABA led to maximum growth value of roots length under 50% and 30% field capacity drought stress in both varieties whereas maximum value of roots dry and fresh weight treated with GABA 2mM and 4mM reported similar results under 50% and 30% field capacity drought stress.

In Mercury variety RWC (Relative water content) is high in 50% and 30% field capacity drought stress at 2mM GABA. However, Scope F1 variety results indicated that RWC (Relative water content) is maximum in 50% field capacity drought stress treated with 2mM GABA whereas in 30% field capacity drought RWC (Relative water content) is most prevalent with 4mM GABA treatment as compared to 2mM GABA. The application of GABA foliar spray evidently lessened the electrolyte leakage in non-stressed and stressed *Capsicum annuum* L. plants. EL (electrolyte leakage) maximum value is reported in 50% and 30% field capacity drought stress in both varieties treated with 2mM GABA application. This analysis of the interplay between drought stress and GABA foliar spray amount discovered that when drought stress worsens, so does electrolyte leakage. Using GABA, on the other hand, inhibits electrolyte leakage.

24

Table 1a. Effect of GABA on roots length, roots fresh weight, roots dry weight, relative water content and electrolyte leakage under induce drought stress.

Varieties	Treatment	Details	RL (cm)	RFW (g)	RDW (g)	RWC (%)	EL (%)
Scope F1	Control	0mM	12.76 ± 0.80	8.06 ± 1.70	2.4 ± 0.74	60.01 ± 3.33	30.47 ± 3.65
	GABA	2mM	15.6 ± 0.2	9.3 ± 1.45	3.49 ± 1.29	66.37 ± 4.45	27.82 ± 2.24
	GABA	4mM	14.34 ± 0.45	8.4 ± 2.09	2.97 ± 0.76	63.15 ± 4.21	29.99 ± 2.00
	50% Field capacity	0mM	11.1 ± 0.17	6.08 ± 0.19	0.90 ± 0.29	48.14 ± 14.66	52.86 ± 5.49
	GABA	2mM	13.1 ± 0.81	7.77 ± 2.43	1.67 ± 0.51	53.26 ± 12.87	45.85 ± 2.77
	GABA	4mM	12.09 ± 1.06	6.70 ± 1.27	1.42 ± 0.36	51.79 ± 5.21	46.41 ± 3.03
	30% Field capacity	0mM	10.13 ± 0.49	3.39 ± 0.77	0.43 ± 0.09	43.35 ± 11.42	59.07 ± 1.83
	GABA	2mM	10.96 ± 0.97	4.44 ± 0.55	1.24 ± 0.14	46.76 ± 7.19	54.28 ± 7.09
	GABA	4mM	11.12 ± 0.42	4.7 ± 1.29	1.20 ± 0.09	47.85 ± 12.43	57.07 ± 7.51

Table 1 (continued)

Varieties	Treatment	Details	RL (cm)	RFW (g)	RDW (g)	RWC (%)	EL (%)
Mercury	Control	0mM	14.56 ± 1.23	10.7 ± 1.22	3.31 ± 0.47	67.20 ± 13.41	33.63 ± 0.80
	GABA	2mM	16.73 ± 1.02	12.16 ± 1.15	3.86 ± 0.23	72.34 ± 13.16	28.02 ± 3.10
	GABA	4mM	14.83 ± 0.93	9.5 ± 0.52	2.96 ± 0.31	70.30 ± 4.00	33.33 ± 5.06
	50% Field capacity	0mM	10.4 ± 1.25	6.15 ± 1.00	0.72 ± 0.39	52.84 ± 5.15	47.50 ± 3.18
	GABA	2mM	13.09 ± 0.76	7.51 ± 0.53	1.56 ± 0.12	56.73 ± 16.39	43.99 ± 4.77
	GABA	4mM	11.40 ± 0.43	6.49 ± 0.97	1.03 ± 0.29	54.66 ± 2.47	44.08 ± 2.95
	30% Field capacity	0mM	9.36 ± 1.28	4.09 ± 0.45	0.36 ± 0.20	43.65 ± 7.04	53.03 ± 2.37
	GABA	2mM	10.36 ± 0.45	5.39 ± 0.78	0.64 ± 0.15	47.59 ± 3.11	49.96 ± 8.41
	GABA	4mM	9.86 ± 0.60	4.88 ± 0.68	0.60 ± 0.04	45.14 ± 6.90	51.04 ± 4.44

Roots length = RL, Roots fresh weight = RFW, Roots dry weight = RDW, Relative water content = RWC, Electrolyte leakage = EL.

3.2. Effect of GABA on shoots length, shoots fresh weight, shoots dry weight and root shoot ratio under induced drought stress.

According to the results of table 3a and 3b, the usage of exogenous GABA foliar spray resulted in maximum values of shoots length, shoots fresh weight, shoots dry weight and RSR (root shoot ratio) under 50 percent and 30 percent field capacity drought stress in both varieties of *Capsicum annuum* L., Scope F1 and Mercury. In both cultivars, the administration of 2mM GABA resulted in the highest value of shoots fresh and dry weight under 50 % and 30 % field capacity drought stress. Similarly, RSR (root shoot ratio) maximum value was reported in 50 % and 30 % field capacity drought stress in both varieties treated with 2mM GABA application. Exogenous treatment of GABA considerably improves growth metrics in *Capsicum annuum* L. under drought stress conferring to this investigation of the interaction between drought stress and GABA foliar spray quantity.

Table 2. Effect of GABA on shoots length, shoots fresh weight, shoots dry weight and RSR under induce drought stress.

Varieties	Treatment	Details	SL (cm)	SFW (g)	SDW(g)	RSR ($g\ g^{-1}$)
Scope F1	Control	0mM	30.13 ± 0.90	27.37 ± 2.10	6.65 ± 0.93	0.37 ± 0.15
	GABA	2mM	34.23 ± 1.05	31.80 ± 2.65	8.11 ± 1.94	0.43 ± 0.14
	GABA	4mM	32.3 ± 1.70	28.18 ± 1.73	6.76 ± 0.99	0.43 ± 0.06
	50% Field capacity	0mM	27.93 ± 1.68	24.08 ± 0.46	4.91 ± 0.98	0.18 ± 0.08
	GABA	2mM	31.03 ± 2.07	28.31 ± 2.74	7.18 ± 0.61	0.23 ± 0.06
	GABA	4mM	29.1 ± 1.3	26.75 ± 1.51	6.52 ± 1.50	0.23 ± 0.12
	30% Field capacity	0mM	25.46 ± 1.58	19.06 ± 1.32	3.41 ± 1.19	0.13 ± 0.01
	GABA	2mM	28.03 ± 2.96	21.84 ± 1.72	4.51 ± 1.01	0.28 ± 0.06
	GABA	4mM	28.93 ± 0.56	20.86 ± 1.55	4.07 ± 0.67	0.30 ± 0.06

Table 2. (Continued)

Varieties	Treatment	Details	SL (cm)	SFW (g)	SDW (g)	RSR (g g^{-1})
Mercury	Control	0mM	33.33 ± 0.86	29.17 ± 1.79	7.50 ± 0.84	0.44 ± 0.10
	GABA	2mM	36.63 ± 1.95	32.4 ± 1.55	9.08 ± 0.86	0.42 ± 0.02
	GABA	4mM	34.33 ± 1.20	30.07 ± 2.13	8.42 ± 1.09	0.35 ± 0.01
	50% Field capacity	0mM	29.92 ± 1.00	24.48 ± 1.33	5.18 ± 0.92	0.13 ± 0.04
	GABA	2mM	32.4 ± 2.19	26.51 ± 1.77	6.36 ± 0.79	0.24 ± 0.02
	GABA	4mM	30.1 ± 1.47	25.82 ± 2.84	6.19 ± 0.96	0.17 ± 0.07
	30% Field capacity	0mM	26.56 ± 0.95	18.19 ± 2.20	3.12 ± 0.11	0.11 ± 0.07
	GABA	2mM	28.33 ± 1.50	18.95 ± 0.97	3.76 ± 1.70	0.21 ± 0.15
	GABA	4mM	27.46 ± 1.20	19.30 ± 1.47	4.20 ± 0.64	0.14 ± 0.02

Shoots length = SL, Shoots fresh weight = SFW, Shoots dry weight = SDW, Root shoot ratio = RSR.

3.4. Regression and correlation study of biochemical and physiological parameters in sweet pepper.

Study of correlation and regression (Table 4) represents the significant and positive relation between chlorophyll “a” of drought stress and Chlorophyll “a” of GABA treated plants which was r = 0.320 and r = 0.702, chlorophyll “b” of drought stress was r = 0.198 and GABA treated was recorded r = 0.446, whereas similar results were determined for Chlorophyll a/b ratio, TCC (total chlorophyll content), CC (carotenoids content), SSC (soluble sugar content), SPC (soluble protein content) CAT (catalase content), APOX (ascorbate peroxidase content). These results clearly demonstrated that GABA treatment significantly increased the biochemical content of *Capsicum annuum* L. The study found that foliar spray had a substantial contribution to the Chloro "a", Chloro "b", Chloro a/b ratio, TCC (total chlorophyll content), CC (carotenoids content), SSC (soluble sugar content), SPC (soluble protein content), of sweet pepper plant. Antioxidant enzymes such as SOD (Superoxide dismutase) and POD (peroxidase activities) in *Capsicum annuum* L also increased, when compared to water deficit stress treatments. Whereas results recorded for TPC (total proline content) were not significant.

Table 3. Regression and Correlation analysis between physiological and biochemical features in *Capsicum annuum* L.

Correlation analysis between drought stress and *Capsicum annum* L.										
Chloro “a”	Chloro “b”	Chloro a/b	TCC	CC	SSC	SPC	TPC	POD	SOD	CAT	APOX
$r=0.320$	$r=0.198$	$r=0.331$	$r=0.391^{***}$	$r=0.221$	$r=0.349$	$r=0.321$	$r=0.301$	$r=0.270$	$r=0.284$	$r=0.471^{***}$	$r=0.484^{***}$
$R^2=0.102$	$R^2=0.039$	$R^2=0.109$	$R^2=0.153$	$R^2=0.049$	$R^2=0.122$	$R^2=0.103$	$R^2=0.091$	$R^2=0.073$	$R^2=0.081$	$R^2=0.222$	$R^2=0.235$
Correlation analysis between GABA and *Capsicum annum* L.											
Chlo “a”	Chlo “b”	Chlo a/b	TCC	CC	SSC	SPC	TPC	POD	SOD	CAT	APOX
$r=0.702^{***}$	$r=0.446^{***}$	$r=0.641^{***}$	$r=0.576^{***}$	$r=0.576^{***}$	$r=0.388^{***}$	$r=0.554^{***}$	$r=0.191$	$r=0.561^{***}$	$r=0.320^{***}$	$r=0.485^{***}$	$r=0.523^{***}$
$R^2=0.492$	$R^2=0.199$	$R^2=0.411$	$R^2=0.332$	$R^2=0.331$	$R^2=0.150$	$R^2=0.307$	$R^2=0.036$	$R^2=0.315$	$R^2=0.102$	$R^2=0.235$	$R^2=0.273$

Chlorophyll a = Chloro “a”, Chlorophyll b = Chloro “b”, Chlorophyll a/b ratio = Chloro a/b, Total chlorophyll = TCC, Carotenoids = CC, Soluble sugar content = SSC, Soluble protein content = SPC, Total proline content = TPC, Peroxidase = POD, Superoxide dismutase = SOD, Catalase = CAT, Ascorbate peroxidase = APOX, * is significant up to $p = 0.05$, Correlation analysis *** is significant up to p: 0.001.

3.5. Analysis of multi-correlated biochemical and physiological parameters in *Capsicum annuum* .L treated with foliar spray

Correlation analysis showed in Table 5, estimated positive correlation between Chloro "a," Chloro "b", Chloro a/b, TCC, CC, SPC and POD. Likewise, an optimistic correlation was witnessed between Chloro "b", TCC and CC. All correlations were significant at $p = .01$ and 0.05. In the same way, an optimistic correlation was detected between Chloro a/b, TCC, CC, SPC and POD, whereas CC showed positive correlation with SSC, SPC, POD, SOD and APOX. Correspondingly, there is positively correlated between SSC, SPC, POD, SOD, CAT and APOX. Similarly, TPC evaluated a positive correlation with POD, SOD, CAT and APOX. Likewise, SPC showed correlation with POD and POD showed a positive correlation with SOD, CAT and APOX. Correspondingly, SOD is correlated to CAT, and APOX, whereas CAT is significantly correlated to APOX.

Table 4. Analysis of correlation between physiological and biochemical components in *Capsicum annuum* L.

Traits	Chloro "a"	Chloro "b"	Chloro a/b	TCC	CC	SSC	TPC	SPC	POD	SOD	CAT	APOX
**Chloro "a"**	1											
**Chloro "b"**	.578*	1										
**Chloro a/b**	.897**	0.172	1									
**TCC**	.717**	.635**	.570*	1								
**CC**	.835**	.614**	.676**	0.453	1							
**SSC**	0.355	0.293	0.269	-0.001	.613**	1						
**TPC**	0.317	0.110	0.305	0.125	0.343	0.433	1					
**SPC**	.736**	0.356	.701**	0.287	.826**	.648**	0.338	1				
**POD**	.703**	0.412	.598**	0.430	.701**	.586*	.780**	.544*	1			

**SOD**	0.443	0.453	0.274	0.181	.584*	.685**	.711**	0.447	.770**	1		
**CAT**	0.277	0.064	0.292	0.135	0.206	.571*	.692**	0.382	.631**	.538*	1	
**APOX**	0.466	0.400	0.331	0.386	.515*	.569*	.574*	0.333	.778**	.567*	.642**	1

Chlorophyll a = Chloro "a", Chlorophyll b = Chloro "b", Chlorophyll a/b ratio = Chloro a/b, Total chlorophyll = TCC, Carotenoids = CC, Soluble sugar = SSC, Soluble protein content = SPC, Total proline content = TPC, Peroxidase = POD, Superoxide dismutase = SOD, Catalase = CAT, Ascorbate peroxidase = APOX, ** correlation is significant at the 0.01 (2-tailed). * Correlation is significant at the 0.05.

3.6. Analysis of Principal components of the biological traits in *Capsicum annuum* L.

The results of Principal component analysis of the biological traits were based on 12 traits and are represented in table 7 below. The first "PC1" accounted for 53.715% of overall variance, which shows a significant correlation with Chloro "a", Chloro a/b, CC, SPC, POD, SOD and APOX predominantly linked with corresponded antioxidant enzymes and osmoprotectants. However, second "PC2" explained 17.047% of complete variance and correlated with TCC, TPC and CAT, growth response parameters. Similarly, "PC3" explained 9.191% of overall variations with significant variables, e.g., Chloro "b" and SPC. Likewise, "PC4" explained 6.721% of variation with significant variable "SSC".

Table 5. Eigen values, individual variance (%), cumulative variance (%), and coefficients of determination of the first three principal components based on biological component correlation matrix.

	Variance %			Components			
Components	Eigen Values	Individual %	Cumulative %	PC1	PC2	PC3	PC4
Chloro "a"	6.446	53.715	53.715	0.844	0.486	-0.078	0.141
chloro "b"	2.046	17.047	70.762	0.569	0.393	0.508	-0.458
Chloro a/b	1.103	9.191	79.954	0.708	0.408	-0.360	0.420
TCC	1.005	8.372	88.326	0.544	0.589	0.443	0.265
CC	0.537	4.479	92.804	0.857	0.299	-0.182	-0.275
SSC	0.359	2.990	95.794	0.698	-0.373	-0.260	-0.428
TPC	0.213	1.772	97.566	0.659	-0.546	0.095	0.270
SPC	0.128	1.071	98.636	0.768	0.183	-0.514	-0.151
POD	0.078	0.653	99.290	0.916	-0.190	0.113	0.151
SOD	0.049	0.412	99.702	0.767	-0.372	0.130	-0.253
CAT	0.034	0.280	99.982	0.614	-0.573	0.018	0.286
APOX	0.002	0.018	100.000	0.749	-0.279	0.329	0.071

Chlorophyll a = Chloro "a", Chlorophyll b = Chloro "b", Chlorophyll a/b ratio = Chloro a/b, Total chlorophyll = TCC, Carotenoids = CC, Soluble sugar = SSC, Soluble protein content = SPC, Total

proline content = TPC, Peroxidase = POD, Superoxide dismutase = SOD, Catalase = CAT, Ascorbate peroxidase = APOX.

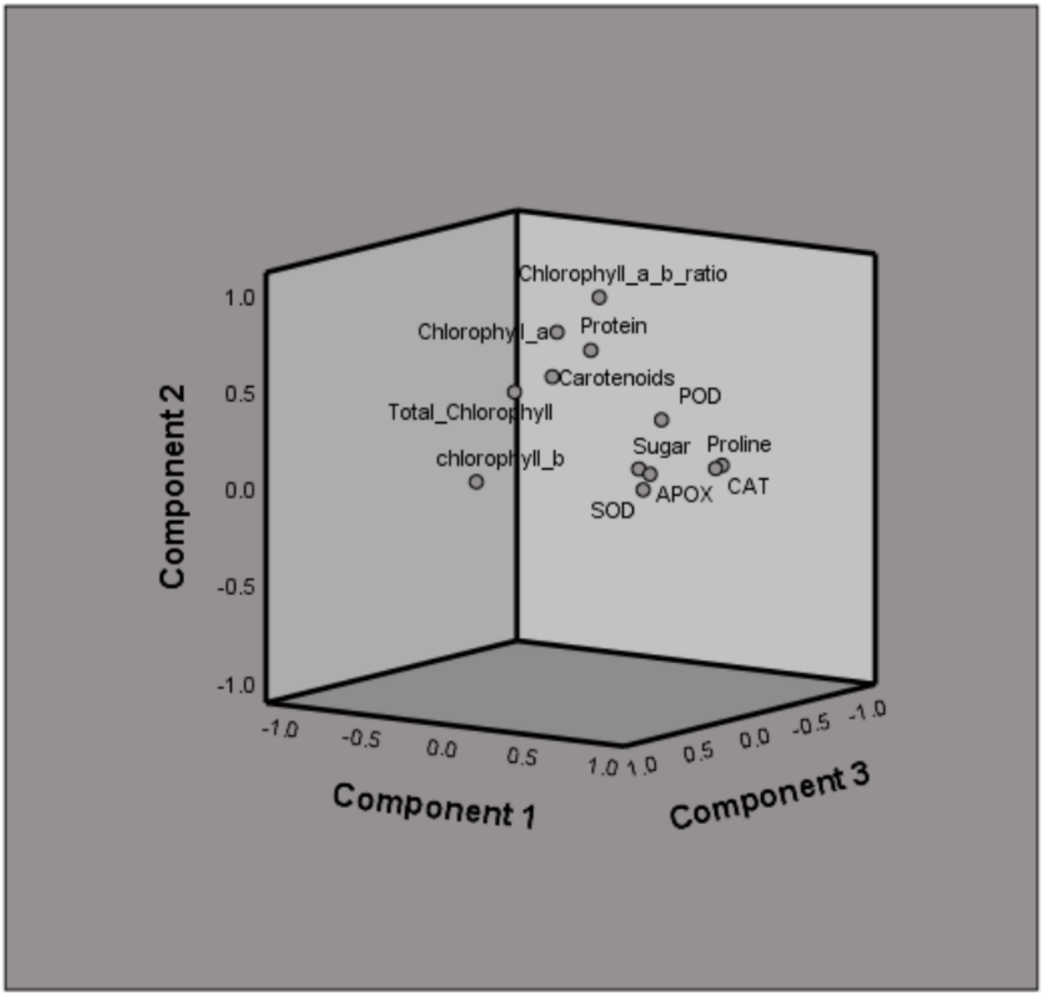

Fig 1. PC1, PC2, and PC3 loading plots in a rotated space.

3.7. Physiological and biochemical characteristics

3.7.1. Impact on Chlorophyll "a" content under drought stress

As revealed in figure 2, the fresh leaves taken from *Capsicum annuum* L. plants grown under drought stress (i.e. 50 % and 30 % field capacity) have significantly lower level of chlorophyll at harvest. The foliar spray has significantly (<.000) augmented the levels of chlorophyll "a" in the fresh leaves of *Capsicum annuum* L. under induced drought stress situations at harvest, regardless of the levels of GABA applied or the cultivars studied in the present research. However, 2mM was more effective on 50 % field capacity drought stress whereas 4mM GABA showed better result on 30 % field capacity drought stress.

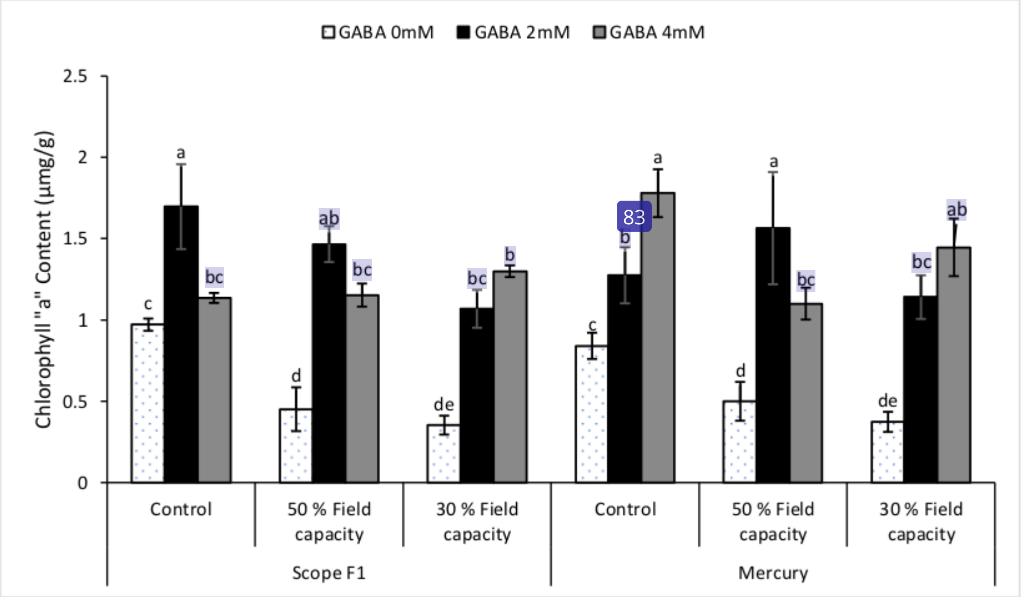

Fig 2. Effect of GABA applied in the concentration of 0mM, 2mM and 4mM on chlorophyll "a" content of *Capsicum annuum* L. under induced drought stress.

3.7.2. Impact on chlorophyll "b" content under drought stress

Figure 3 represented that drought stress significantly declined the chlorophyll "b" content in the leaves of the *Capsicum annuum L.* plants. The foliar application significantly (<.02) enhanced the chlorophyll "b" content of the fresh leaves of *Capsicum* plants treated GABA irrespective of the levels of application, indicating that foliar spray can increase the activity of chlorophyll under water stress conditions.

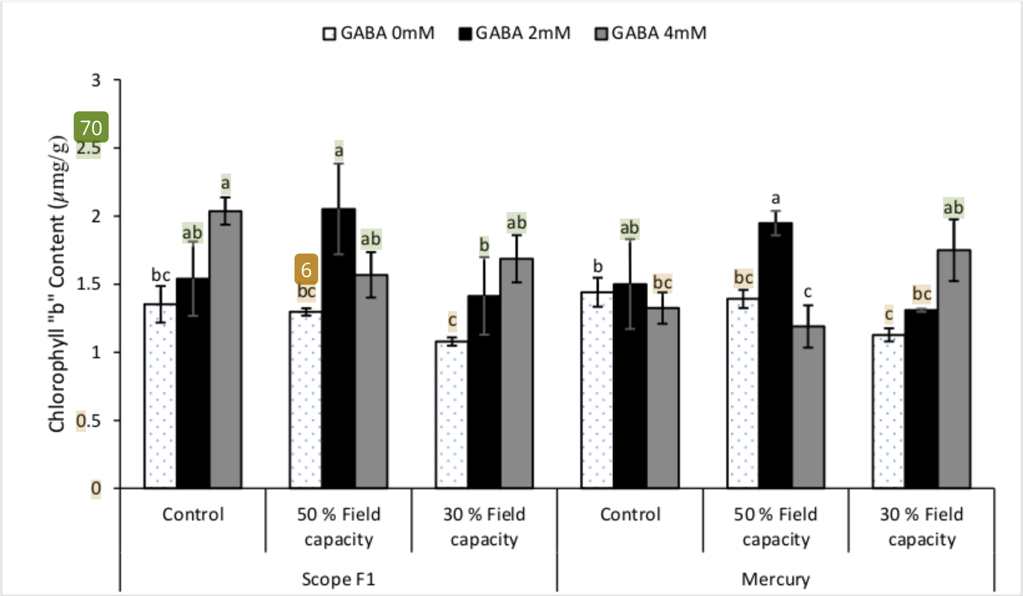

Fig 3. Effect of GABA applied in the concentration of 0mM, 2mM and 4mM on chlorophyll "b" content of *Capsicum annuum* L. under induced drought stress.

3.7.3. Impact on chlorophyll "a/b" ratio content under drought stress

A change can be seen in the pattern of Chlorophyll a/b ratio content shown in Figure 4. This revealed that chlorophyll "ab" ratio content was highest in variety Scope F1 and Mercury cultivar under 50 % field capacity and 30 % field capacity drought showing an upsurge in the chlorophyll a/b ratio content under high GABA treatment of both 2mM and 4mM concentration. This indicated that foliar spraying of growth regulators increases the plant's ability to overcome harsh stress conditions and this inherited potential increased by synthesizing chlorophyll more rapidly and efficiently when the species is exposed to stress.

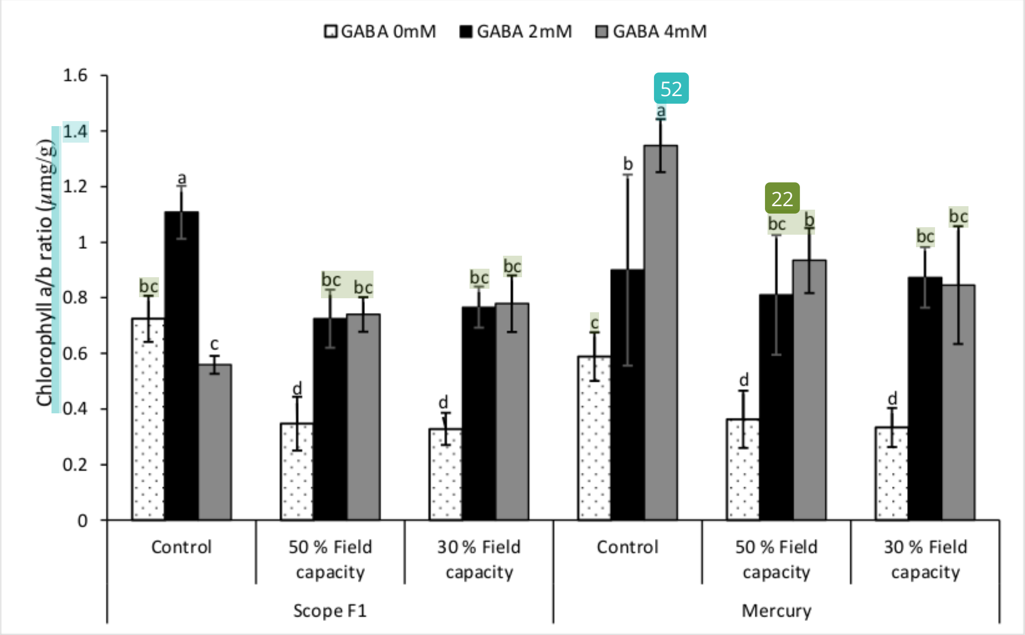

Fig 4. Effect of GABA applied in the concentration of 0mM, 2mM and 4mM on chlorophyll a/b ratio content of *Capsicum annuum* L. under induced drought stress.

3.7.4. Impact on Total chlorophyll content under drought stress

As shown in figure 5, the fresh leaves of *Capsicum annuum* L, plants were significantly (<.007) affected by the water deficiency stress resulting in lower total chlorophyll content. However, the application of GABA at 2mM and 4mM levels showed significant increase in the total chlorophyll content (TCC) of the leaves of sweet pepper plants irrespective of the cultivar. The GABA application of 2mM showed much better results on 50 % field capacity drought stress whereas 4mM GABA was more effective on control and 30 % field capacity drought stress in both varieties.

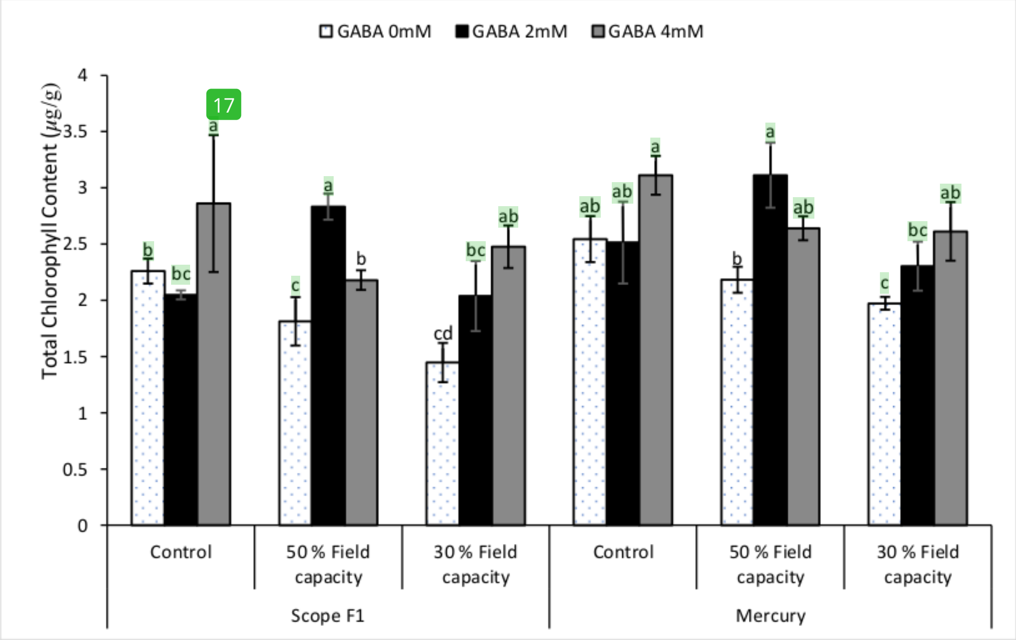

Fig 5. Effect of GABA applied in the concentration of 0mM, 2mM and 4mM on Total Chlorophyll content of *Capsicum annuum* L. under induced drought stress.

3.7.5. Impact on Carotenoids content under drought stress

Significant differences ($p > .05$) were reported among different amount of foliar spray treatments in both cultivars. Carotenoids content (CC) of plant foliar material (Figure 6) was highest under the 2mM GABA treatment in control and 50 % field capacity drought plants whereas for 30 % field capacity drought 4mM GABA treatment showed highest carotenoids content for the Scope F1 variety. Similarly, in Mercury cultivar the carotenoids content showed significance in control and 50 % field capacity drought treated with 2mM-GABA, however 4mM-GABA treatment showed highest carotenoids content for 30 % field capacity drought plants of the same variety ($p < .000$).

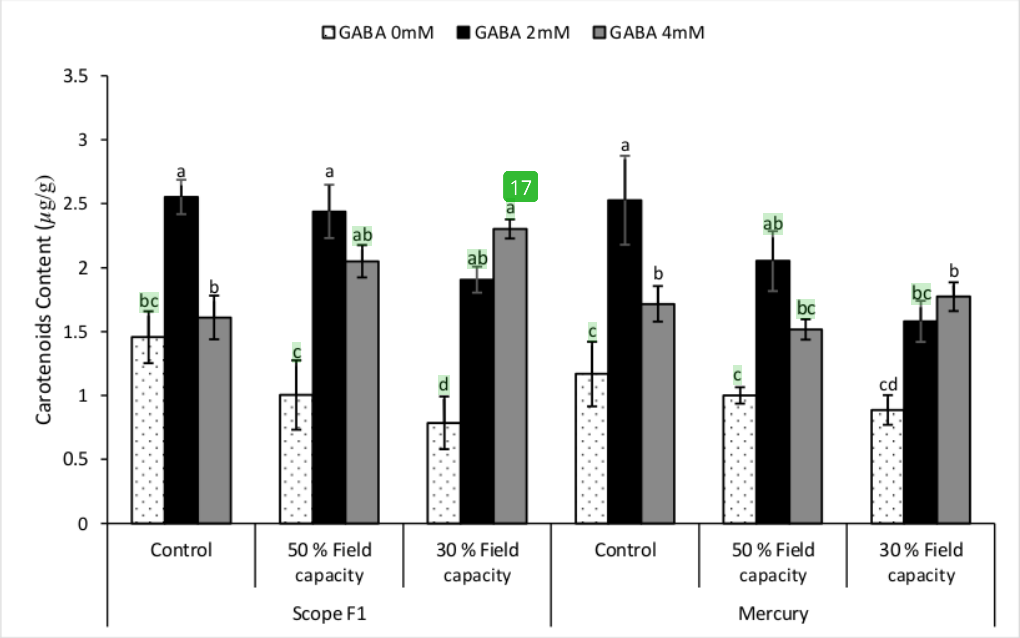

Fig 6. Effect of GABA applied in the concentration of 0mM, 2mM and 4mM on Carotenoids content of *Capsicum annuum* L. under induced drought stress.

3.7.6. Impact on Soluble sugar content under drought stress

Growing *Capsicum annuum* L. plants under drought stress (50 % and 30 % field capacity) conditions resulted in lower levels of soluble sugar content (SSC) in the leaves of both the cultivars examined as compared to control, in the present study (figure 7). Foliar application of 2mM and 4mM GABA significantly (<.004) amplified the levels of sugar content in the leaves of both the cultivars under both water stress conditions (50 % and 30 % field capacity drought). This indicated that growth regulator (especially GABA 2mM) proliferates the plant's ability to overcome harsh stress conditions and this inherited potential increased by synthesizing osmoprotectants more rapidly and efficiently when the species is exposed to drought stress. Both varieties were tolerant in the GABA treated plants perhaps to the resistance mechanism of a broad-spectrum stress resistance being unlocked to survive under various biotic as well as abiotic stresses.

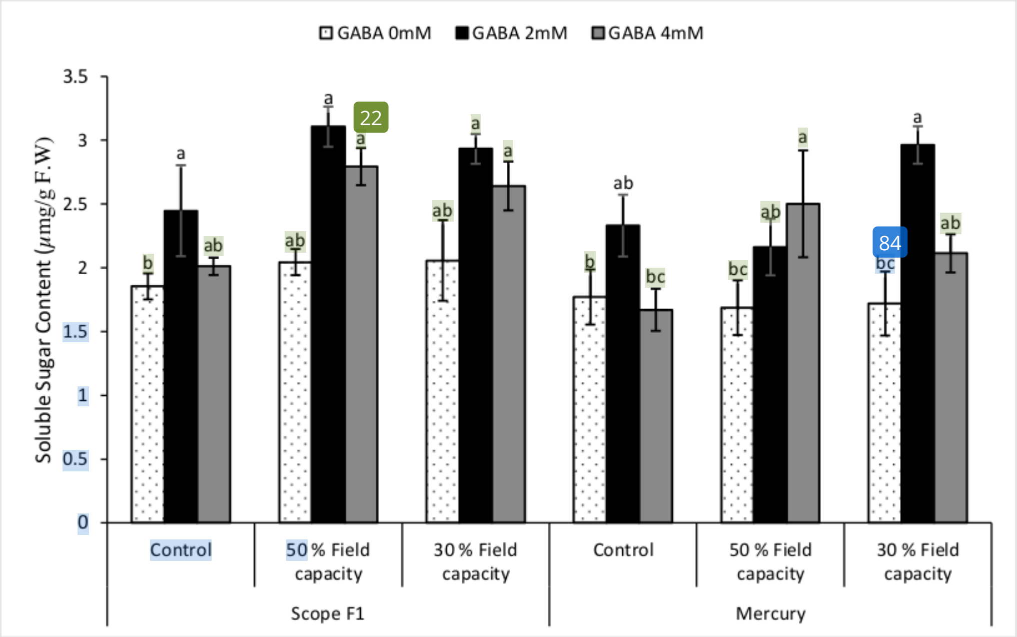

Fig 7. Effect of GABA applied in the concentration of 0mM, 2mM and 4mM on Sugar content of *Capsicum annuum* L. under induced drought stress.

3.7.7. Impact on Total proline content under drought stress

The fresh leaves of *Capsicum annuum* L. showed a decrease in the total proline content (TPC) when exposed to drought stress in both genotypes (Scope F1 and Mercury) investigated in the study. Figure 8 revealed that maximum proline content was reported when the plants were treated with foliar spray of GABA 2mM and 4mM. However foliar spray of 2mM GABA showed much better results in increasing the proline content of both the varieties of *Capsicum annuum* L. plants as compared to 4mM GABA under induced drought stress conditions. (<.002)

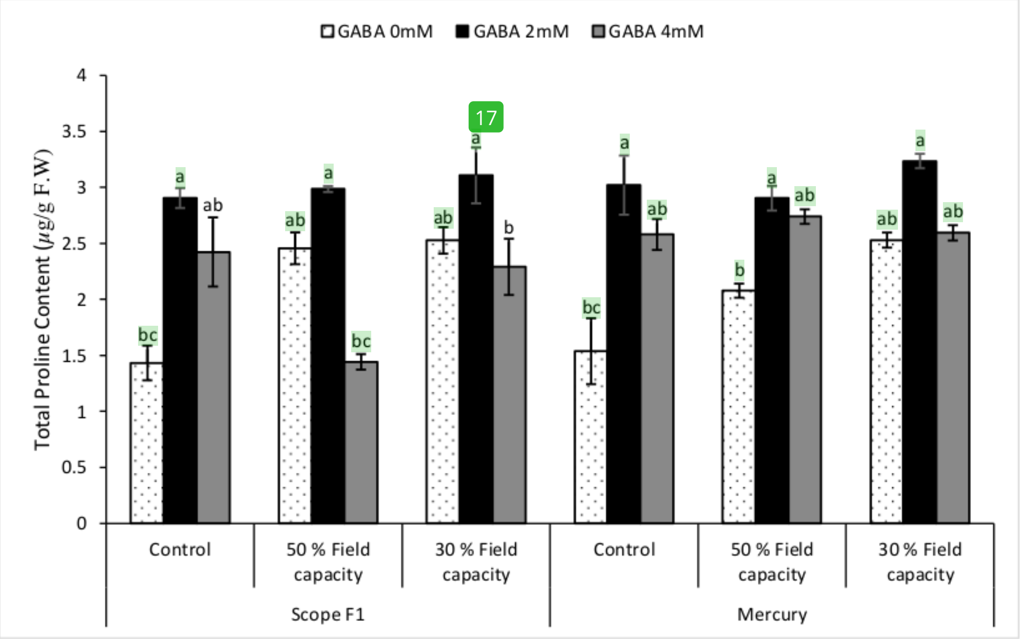

Fig 8. Effect of GABA applied in the concentration of 0mM, 2mM and 4mM on Proline content of *Capsicum annuum* L. under induced drought stress.

3.7.8. Impact on soluble protein content under drought stress

The fresh leaves from sweet pepper plants grown under 30 % field capacity drought stress conditions exhibited significantly lower levels of soluble protein content (SPC) as compared to those taken from plants grown under 50 % field capacity drought stress irrespective of the cultivars used (figure 9). The foliar application of GABA (especially 2mM) significantly (<.001) improved the levels of soluble protein content in the fresh leaves of *Capsicum annuum* L. plants under drought stress (50 % and 30 % field capacity) regardless of the genotypes investigated.

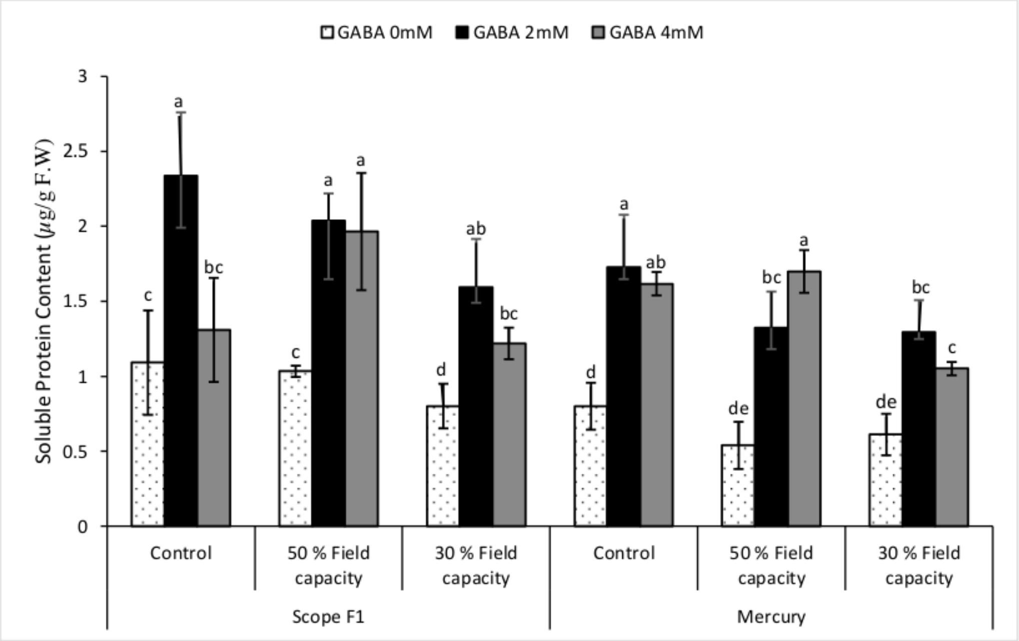

Fig 9. Effect of GABA applied in the concentration of 0mM, 2mM and 4mM on Protein content of *Capsicum annuum* L. under induced drought stress.

3.7.9. Impact on Peroxidase content under drought stress

POD (peroxidase) are vital antioxidant defensive enzymes shielding the plants from adverse effect of drought stress, by the conversion of O_2 to H_2O_2. As shown in figure 10, the POD content in the leaves of *Capsicum annuum* L. is lower under drought stress in both varieties. The foliar spray treatment of GABA at different levels (i.e., 2mM and 4mM) has significantly (<.000) increased the POD content in the leaves of sweet pepper plants irrespective of the genotypes investigated. However, 2mM GABA showed slightly better results as compared to 4mM GABA under both water deficient conditions irrespective of the cultivars studied.

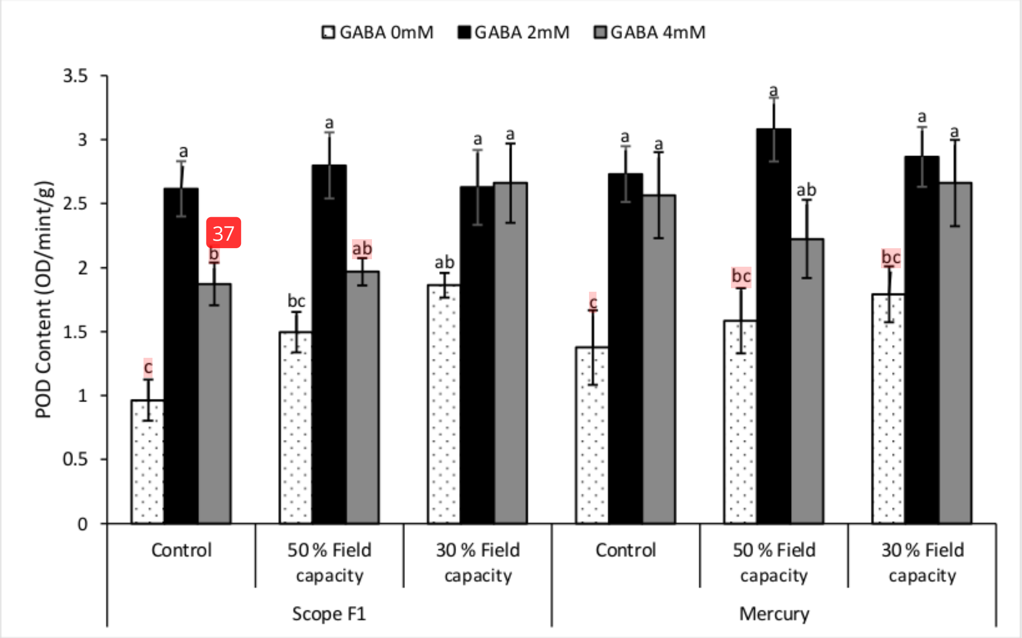

Fig 10. Effect of GABA applied in the concentration of 0mM, 2mM and 4mM on Peroxidase content of *Capsicum annuum* L. under induced drought stress.

3.7.10. Impact on Superoxide dismutase content under drought stress

SOD (superoxide dismutase) is another important antioxidant defense enzyme that protects plants from the harmful effects of drought stress. The conversion of O_2 to H_2O_2 and the breaking of H_2O_2 is increased during drought stress, resulting in an increase in SOD concentration. Figure 11 revealed that application of foliar spray of 2mM-4mM GABA significantly (<.001) increased the SOD content in the fresh leaves of sweet pepper plants under drought stress in both cultivars. However, 2mM GABA showed considerable enhanced results as compared to 4mM GABA under both conditions of drought stress (50 % and 30 % field capacity) irrespective of the cultivars investigated.

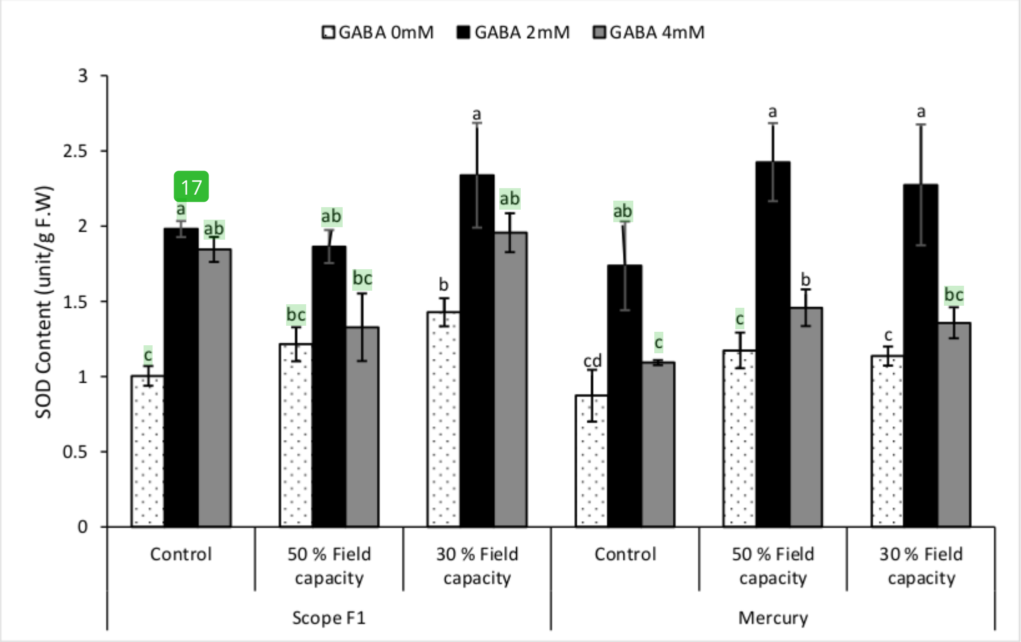

Fig 11. Effect of GABA applied in the concentration of 0mM, 2mM and 4mM on Superoxide dismutase content of *Capsicum annuum* L. under induced drought stress.

3.7.11. Impact on Catalase content under drought stress

As shown in figure 12, leaves of *Capsicum annuum* L. plants showed an increase in CAT (catalase) content, treated with 2mM GABA under both stress conditions (50 % and 30 % field capacity drought) in Scope F1 variety. Whereas in Mercury variety 4mM foliar spray treatment of GABA showed significance under control and 50 % field capacity drought. However, for 30 % field capacity drought 2mM GABA treatment was more effective (p <.000)

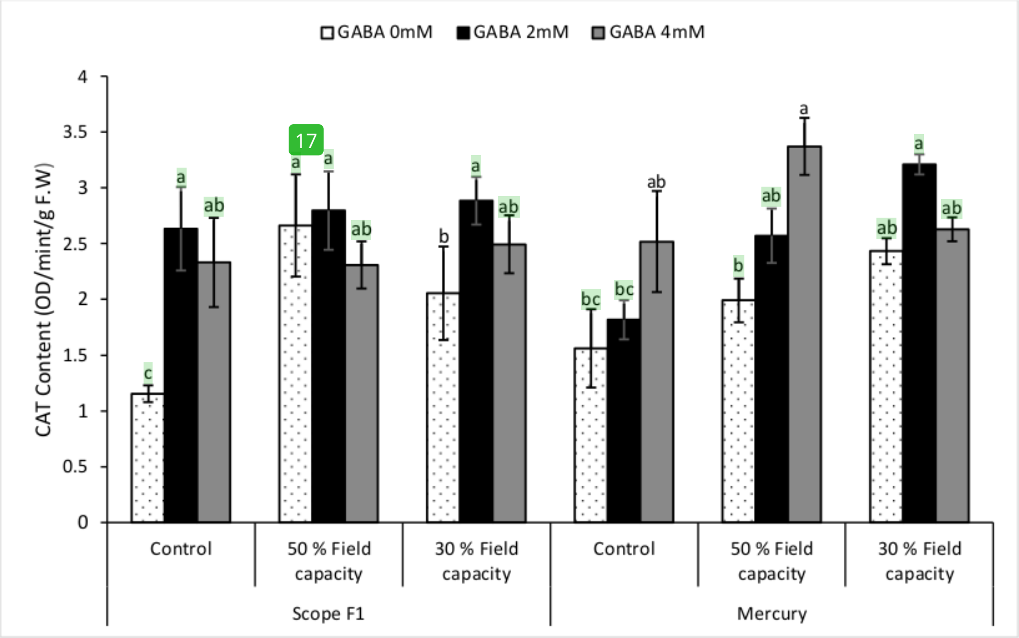

Fig 12. Effect of GABA applied in the concentration of 0mM, 2mM and 4mM on Catalase content of *Capsicum annuum* L. under induced drought stress.

3.7.12. Impact on Ascorbate peroxidase content under drought stress

Results in Figure 13 also indicated that foliar spray treatment showed positive effect on APOX (ascorbate peroxidase) content in both cultivars (i.e. Scope F1 and Mercury) reporting tolerance to drought stress due to high antioxidant defense mechanism by reducing ROS formation. However, foliar spray of 2mM GABA was more effective in Scope F1 variety as compared to Mercury variety where 4mM GABA showed significant results regardless of the drought conditions the plants were exposed ($p < .004$).

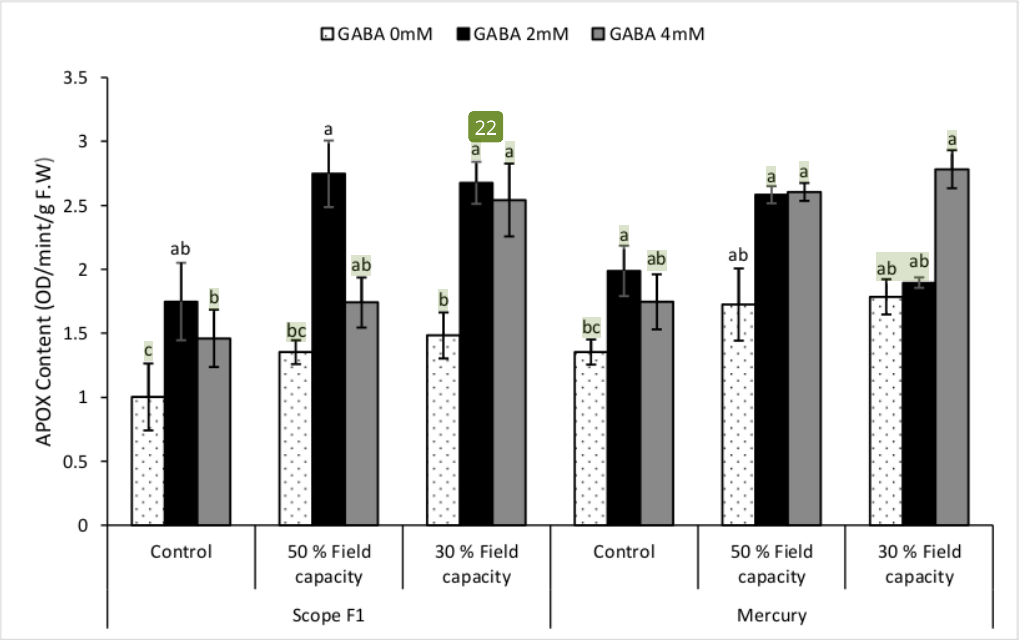

Fig 13. Effect of GABA applied in the concentration of 0mM, 2mM and 4mM on Ascorbate peroxidase content of *Capsicum annuum* L. under induced drought stress.

4. Discussion

Drought is a global problem, and a considerable amount of cultivated land is under drought stress to varying degrees. Drought is the foremost limiting factor for optimum plant development and high yield production, resulting in water shortages, low air humidity, and high temperatures. Plant development was clearly limited under water scarcity stress circumstances owing to a variety of changes in the physiological as well as biochemical processes (Kosar *et al*., 2015). Drought stress can cause significant reductions in growth and development in a wide array of economically vital agricultural crops (Tesfamariam *et al*., 2010). ROS (reactive oxygen species) is often produced by cellular processes such as photosynthesis and respiration in most plants, particularly under situations of diverse environmental challenges (Tripathy and Oelmuller, 2012). ROS (reactive oxygen species) and Free radicals hinders proliferation and cell division, resulting in cell's demise (Dolatabadian *et al*., 2009).

The work of Singh and Roychoudhury (2020) showed that GABA improves the level of numerous osmolytes as well as antioxidants in plants, which ultimately helps to decrease the level of ROS (reactive oxygen species) and MDA (Malondialdehyde) in addition reduction also occurs in membrane damages. Bashir *et al*. (2021) verified that the use of γ-aminobutyric acid foliar spray enhanced the vegetative development and considerably enhanced the levels of free plastid pigments, amino acids, as well as the relative water content and antioxidant enzymes in carrots grown under field capacity of 50% as related to controlled conditions. In addition, GABA usage also lessened MDA (melondialdehyde) and EL (electrolyte leakage) of ions in carrot leaves.

GABA (γ-aminobutyric acid) lessens constrains of the mitochondrial and photosynthetic activities and the rate of lipid peroxidation to help increase water scarcity forbearance in *Piper nigrum* L. throughout the osmotic stress (Vijayakumari and Puthur, 2016). GABAs Exogenous treatment has also been shown to increase heat tolerance in rice (Oryza sativa) (Nayyar *et al*., 2014). The statement of Li *et al*. (2016) also shows the significance of GABAs on *Agrostis stolonifera* (creeping bentgrass) refining its tolerance to drought stress. Research have revealed that GABAs alleviate cold damage in many plants such as banana (Wang *et al*., 2016) tomato (Malekzadeh *et al*., 2014) *Cucurbita pepo* (Palma *et al*., 2019), *Cucumis sativus*, (Malekzadeh *et al*., 2017) and peach (Bustamante *et al*., 2016).

Some reviews have shown that drought stress has a negative impact on some factors such as fresh weights and dry weights of plant for example *Helianthus annuus* (Abdel Razik *et al*., 2021; Hossain *et al*., 2010) and *Brassica Napus* (Tesfamariam *et al*., 2010), which can be

clarified by variations that occur at the bio-physiological level of the plants as a result of water scarcity (Ashraf *et al.*, 2011; Shafiq *et al*., 2014). H_2O as we all know is one of the supreme chemicals in every plant since it may be employed as a product, reactant, solvent as well as transporter. For the transport of nutrients in plants water plays a crucial part (Furlan *et al.*, 2013). Additionally, in *Capsicum annuum* L. plants weight loss may be related to a decrease in turgor pressure (Ahmed *et al.*, 2014). This corresponds to the findings of our experiment.

Drought stress (i.e., 30% FC and 50% FC in the current study) significantly ($p \leq 0.001$) reduced the fresh as well as dry weight and length in both cultivars of sweet pepper. It exhibited an additional disastrous impact on shoots length than on roots length (Table 2a and 2b). Scarcity of water has a dramatic influence on the growth characteristics of sweet pepper plants, as demonstrated by our research. Plant growth characteristics such as roots length, fresh and dry weight as well as shoots length, fresh and dry weight and RSR (root shoot ratio) were all lowered by drought stress (Table 2a, 2b, 3a and 3b). GABA therapy (foliar spray) was used in the current study at varying concentrations (2mM and 4mM). Regardless of the dose of GABA used in the current study, foliar application of GABA increased ($p \leq 0.001$) the vegetative development of sweet pepper plants grown under drought-stressed circumstances.

Nonetheless, the foliar use of 2mM GABA spray exceeded other treatments in refining the roots and shoots length, fresh and dry weights and RSR (root shoot ratio) of *Capsicum annuum* L. plants grown under 50% and 30% field capacity conditions, regardless of the cultivars. The effects of GABA on sweet pepper plants were successful in both the controlled group and under stressed settings in the current study. As an outcome of the collected results, the exogenous administration of GABA boosted all growth factors (Tables 2a, 2b, 3a and 3b). The current study's findings are congruent with those of (Vijayakumari and Puthur, 2016), who discovered that using GABA mitigated the negative impacts of water deficiency stress on developmental constraints of *Piper nigrum* L. (black pepper). These outcomes were also in approval with those currently stated in muskmelon (Jin *et al.*, 2019) *Oryza sativa* (Ma *et al.*, 2016) and *Trifolium repens* L. (Cheng *et al.*, 2018) grown under saline soil, *Brassica juncea* (Mahmud *et al.*, 2017) under heavy metals grown under ammonium toxicity.

The RWC (relative water content) of plant tissues is a measure used to evaluate metabolic activity (Soltys-Kalina *et al.*, 2016). Drought stress conditions significantly decreased RWC (relative water content) in this research (Table 2a and 2b). However, the usage of GABA (especially 2mM) lead to an upsurge in RWC (relative water content) (Table 2a and 2b). The results of the analysis of GABA's effect on *Capsicum annuum* L. under water scarcity stress exhibited that the optimistic impact of GABA in the studied traits can be credited to

refining the GABA effectiveness in water absorption, which can eventually increase the turgor pressure and preserve a water balance in the tissues of *Capsicum annuum* L. plant. These findings were congruent with those previously published in carrot plants by Bashir *et al*. (2021), who discovered that using GABA mitigated the negative effects of water stress on carrot's RWC (relative water content).

One of the criteria used to calculate the damaged membrane index is electrolyte leakage. The peroxidation of saturated fatty acids in the phospholipid membrane explains why EL (electrolyte leakage) occurs during stress. As a result, electrolyte leakage may be computed as a standard for evaluating bio-membrane degradation (Lukatkin and Anjum, 2014). The information in Tables 2a and 2b revealed that leakage of electrolytes rises in reply to increased water deficiency stress. According to Hemmati *et al*. (2018), drought stress can cause cell membrane damage in pot marigold plants. The current study found that GABA (particularly 2mM) decreased the detrimental effects of ROS (reactive oxygen species) and boosted plant tolerance to drought stress.

Exogenous GABA administration has also been shown to relieve the detrimental outcomes of drought stress by enhancing membrane integrity of cells in perennial ryegrass (Li *et al*., 2016; Krishnan *et al*., 2013) muskmelon (Jin *et al*., 2019), *Trifolium repens* L. (Yong *et al*., 2017), and *Agrostis stolonifera* (Li *et al*., 2016). These verdicts imply that GABA may have a role in protecting the membrane of sweet pepper leaves cells from the detrimental impacts of water scarcity stress, most likely through an anti-oxidative mechanism that led to lesser level of EL (electrolyte leakage). A larger investigation might assist to demonstrate GABA's protective role against water deficiency stress at the cellular level.

Drought is one of the most critical factors affecting *Capsicum annum* L. (sweet pepper) growth, inhibiting plant development in its early stages. Drought stress is known to cause an upturn in respiration, a decrease in cell membrane injury and photosynthesis in higher plants (Wang *et al*., 2009; Turkan *et al*., 2005). Furthermore, ROS (reactive oxygen species) production as a result of water scarcity lowers chlorophyll as well as carotenoids concentration (Chéour *et al*., 2014). Conferring to the findings of this investigation, the decrease in chlorophyll "a" and "b" concentration in *Capsicum annuum* L. might be ascribed to the unfavourable impacts of water deficiency stress. Reduced chlorophyll concentration is a frequent result of water deficiency stress in *Helianthus annuus* (Ebrahimian and Bybordi, 2012). Furthermore, the plant's capacity to absorb water is hampered by a lack of available water and salt buildup around the root region causes osmotic stress and ion toxicity, which increases the danger of plant damage (Ebrahimian and Bybordi, 2011).

The correct estimate of leaf photosynthetic pigments is a crucial component in monitoring plant stress, which varies between plant species and is affected by CO_2 in the air, light intensity, soil water content, and temperature. The chlorophyll content and carotenoids content of leaves obtained from the *Capsicum annuum* L. plant in our research work is much decreased when subjected to drought stress (50% and 30% field capacity). GABA foliar treatment substantially improved the quantity of chlorophyll and carotenoids content in *Capsicum annuum* L. fresh leaves at harvest.

The results in Figs. 2, 3, 4, 5, and 6, demonstrated that high chlorophyll "a" and "b" and TCC, a/b ratio and carotenoids contents were measured under induced drought stress plants treated with GABA-2mM and 4mM in both varieties of *Capsicum annuum* L, Scope F1 and Mercury were significant at *p*.001, whereas untreated plants at 50 % field capacity and 30 % field capacity drought in both varieties clearly indicated that drought greatly reduced chlorophyll and carotenoids content of the plants, resulting in low photosynthetic rate.

These findings show that in the planned experiment, drought can limit growth responses by degrading photosynthetic pigments, which can be preserved by foliar spraying (especially GABA 2mM and 4mM), which can alter ion homeostasis as GABA is a key metabolite manufactured in answer to drought stress. Adjusting the osmotic potential of agricultural plants by foliar spraying with a helpful growth regulator might be regarded a good approach for the upcoming climate change, which would increase drought levels in the region and reduce crop productivity. Our findings are consistent with earlier research in a variety of field and horticultural crops (Li *et al.,* 2016; Rezaei *et al.,* 2018; Liu *et al.,* 2019; Li *et al.,* 2019; Jisha and Puthur, 2016).

The pattern of change in soluble sugar (SSC) content under drought stress was significant in both sweet pepper varieties, indicating that under stress conditions, an osmoprotective mechanism initiated in the plant for the production of osmoprotectants, such as sugar and proline, to protect against the adverse effects of the stress situation. The present findings reveal an increased sugar content in leaves of water-stressed *Capsicum annuum* L. plants subjected to GABA (especially 2mM) application (figure 7).

According to Wattanakulpakin *et al*. (2012)'s estimation based on prior study, sugar content is required for cowpea seedling germination. Under water shortage stress, it appears that a proliferation in osmolytes i.e., proline and sugars, is a mechanism in *Capsicum annuum* L. for balancing water potential in the plants. Under drought stress, an upsurge in sugar concentration may occur as a method of osmotic potential correction (Ebrahimian and Bybordi, 2012). The present findings revealed an upsurge in the accumulation of soluble sugars during

GABA supplementation in water-stressed *Capsicum annuum* L. plants. Thus, in the present work, GABA signaling during water-stress is expected to regulate carbohydrate metabolism. In summary, it may be inferred that osmotic adjustment via accumulation of amino acids or sugars play a critical part in plant confrontation or tolerance during water deprivation stress situations.

Furthermore, carbohydrates and proline have a significant influence on resistance to water stress in plants. Amino acid such as proline is one of the utmost common amino acids, operating as a well-matched solute for stressful situations (Hayat *et al.*, 2012). Proline protects plants against abiotic stressors, and its content fluctuates according to the degree of the stress in the plant species (Pirzadah *et al.*, 2019; Ahanger *et al.*, 2017; Soliman *et al.*, 2019). As a result, it is not unexpected that proline buildup will rise during water deficit situations. In the present work, GABA application seems to exert marginal influence on these plant growth parameters in well-watered conditions. Water stress in *Capsicum annuum* L. triggers the accumulation of the proline and soluble sugars (Figure 7 and 8). Present evidence is in congruence with earlier reports on the part of GABA in the regulation of osmolytes namely, proline and soluble sugars (Seifikalhor *et al.*, 2020).

Furthermore, GABA's use further enhances the accumulation of osmolytes thus suggesting its role in osmotic tolerance in water-stressed plants (present work). GABA application (especially 2mM) is affective in imparting osmotic tolerance in water-stressed plants. GABA application in drought-stressed white clover plants lead to a higher buildup of GABA endogenously which was accompanied by a positive upregulation in proline metabolism and enzymes of GABA-shunt pathway (Yong *et al.*, 2017). Thus, earlier findings on GABA-induced positive regulation of proline metabolism provide supporting evidence to our present findings. GABA application (especially 2mM) resulted in a surge in proline accumulation (Figure 8) in water-stressed *Capsicum annuum* L. (present work).

GABA is also known to be an effective regulator of carbon metabolism and modulates the activity of NADP-dependent isocitrate dehydrogenase (NADP-ICDH) and phosphoenolpyruvate carboxylase (PEPCase) (Barbosa *et al.*, 2010). Upregulation of GABA shunt has been attributed to GABA-induced accumulation of TCA cycle intermediates (Araújo *et al.*, 2010). Furthermore, whereas *Thymus vulgaris* L., growth reduced with cumulative water shortage stress, proline and thymol buildup considerably augmented (Babaee *et al.*, 2010), which is consistent with the results of our research for *Capsicum annuum* L.

Similarly, water deficit stress reduces the activity of numerous enzymes and causes a net upsurge in free amino acids, which slows down synthesis of protein and/or activation and

accelerates protein breakdown in plants. The minimum protein content of Scope F1 and variety Mercury treated with 2mM and 4mM GABA at a level of induced drought of 50% and 30% field capacity at *p*.05 described that this comeback of crop plants rests on the intensity and duration of the drought, plant species, stage of development, and also the growing season (Figure 9). However, in the current study, the lowest drought level effect was mitigated by foliar spray methods, demonstrating that total soluble protein content did not reduced, implying that proteins linked with antioxidant enzymes have increased.

According to Krishnan *et al*. (2013) plants may defend themselves from oxidative damage produced by ROS via an antioxidant system that is either enzymatic or non-enzymatic. Accumulation of reactive oxygen species is then neutralized by antioxidant enzymes to protect plants from oxidative damage under stress conditions, however this method varies greatly between plant species and even cultivars of the same species.

Water deficiency during drought stress is known to trigger both enzymatic and non-enzymatic antioxidative defense thus leading to redox homeostasis (Laxa *et al*., 2019). In the current research, our results depict that water-stress resulted in a noticeable proliferation in the activity of POD, SOD, CAT and APOX (Figure 10, 11, 12, 13). Under water deficiency stress, there is an increase in proline buildup as well as CAT and APOX activities in black cumin (Balouchi *et al*., 2012). A similar trend was observed for all the four enzymes thus suggesting their integrative role in regulating water stress tolerance in *Capsicum annuum* L. Exogenous application of GABA positively upregulated the activity of all the four antioxidant enzymes.

The peroxidase and superoxide content (figure 10 and 11) showed a significant increase under stress conditions treated with 2mM GABA under both 50 % and 30 % field capacity drought stress indicating that foliar spray treatment can reduce the negative impact of low level of water stress and can be tolerated by both the species. The water stress effect was quenched via foliar spray treatments in the present study meaning that SOD content did not decline indicating that SOD is associated with antioxidant enzymes and showed an increase. This clearly revealed that increased POD and SOD activity might be due to increased H_2O_2 production and consumption under stress condition.

Results in Figures 12 and 13, also indicated that foliar spray treatment showed positive effect on both varieties declaring tolerance to drought stress due to high antioxidant defense mechanism by reducing ROS formation. The foliar spray of 2mM GABA was more effective in increasing the CAT activities (figure 12) in sweet pepper grown under water shortage circumstances as compared to other levels, regardless of the genotypes used, while foliar use

of 4mM GABA spray was more effective in Mercury variety and 2mM GABA was more successful in Scope F1 variety for APOX activities (figure 13).

Altogether, the actions of antioxidative enzymes proliferates in response to reduce the harmful effects of reactive oxygen species generated in excess during water scarcity stress. The activity of all antioxidant enzymes investigated in this study were significantly elevated in the foliar parts of sweet pepper grown under water-stressed circumstances.

Previous reports have established the role of GABA in the modulation of antioxidative defense in Brassica and *Zea sp*. (Mahmud *et al*., 2017). GABA-mediated surge in the activity of SOD, APOX and dehydroascorbate reductase (DHAR) is accompanied by reduced lipid peroxidation in salinity and alkalinity-stressed musk-melon plants (Jin *et al*., 2019). In support to earlier findings, present investigation on *Capsicum annuum* L. plants reveals GABA-induced upregulation of antioxidative enzymes to be accompanied by reduced lipid peroxidation.

Grounded on the outcomes of this research, it seems that use of GABA foliar spray (especially 2mM) increases *Capsicum annuum* L. tolerance to water deficiency stress by boosting chlorophyll concentration, water absorption, and photosynthetic effectiveness, antioxidant enzymes and osmoregulators. The positive impacts of GABA treatment on plants answer to water deficiency stress have also been found in other crops grown with various circumstances. According to the findings of Wang *et al*. (2009), GABA might increase wheat lenience to high temperature stress by foraging reactive oxygen species using antioxidative enzymes, refining membrane stability, and regulating nitrogen and carbon remobilization.

In addition, Li *et al*. (2016) observed that under high temperature stress, GABA's administration might enhance the accumulation of amino acids, organic acids, and sugars in *Agrostis stolonifera* L. GABA administration has been linked to an upsurge in the development and net photosynthesis of maize seedlings (Li *et al*., 2016). In summary, the current study's results are consistent with previous reports, showing that exogenous administration of GABA might be considered as a feasible management strategy for preserving *Capsicum annuum* L. production and growth during drought stress circumstances.

5. Conclusions

Conferring to the findings of the proposed experiment, global climate change would cause severe drought, affecting the yield of our most valued crops in Pakistan, including *Capsicum annuum* L., as well as negatively impacting ecological fitness. It was discovered that various foliar spraying techniques that are deemed economically inexpensive, affordable, and ecologically acceptable are more noteworthy for enzymatic antioxidant activities at the vegetative stage and beginning several metabolic processes in the plant, as well as lowering germination time. The current study's agronomic, physiological, and biochemical analyses revealed that changes in plants due to foliar application provide new knowledge that may be useful in improving the understanding of plant's drought tolerance and will reduce the deleterious effect of the region's upcoming drought threat due to global variation. To differentiate all foliar treatment methods in pepper cultivars, more investigation with other compounds such as mannitol, α-Tocopherol, ascorbic acid, and other growth regulators is needed. The botanical exploration community has gathered a relatively complete understanding of the physiological systems that permit productivity preservation in response to water scarcity stress across many decades. As a result, the groundwork for the production of exceptional crop types adapted to climate change has been laid. To address the food and fuel security issues of a growing population, biotechnology, and breeding efforts to harness these mechanisms must be increased in the upcoming decade. Regardless of this, individuals apprehensive with crops development must proceed with prudence and guarantee that potentially harnessed methods are practical in the situation of Pakistan's continuously shifting environment. Furthermore, in order to keep our farmland fertile and increase healthy growth rates in Pakistan, we must acquire proper irrigation methods to reduce the risk of increasing drought in the region in the future, as well as initiate immediate adaptive procedures proposed by the current study and further interrelated findings of the scholars.

References

Saqib, M. and Anjum, M. A. (2021). Mitigation of climate change effect in sweet pepper (*capsicum annuum* l.) through adjustment of planting time. *Pakistan Journal of Agricultural Sciences*, *58*(3).

Janeeshma, E., Mirshad, P. P. and Puthur, J. T. (2016). Dynamics of Arbuscular Mycorrhizal Development in Relation to Various Growth Stages of Zea mays L. *Climate change and its impact on water resources of Kerala*, 1975-1979.

Yeo, A. (1998). Predicting the interaction between the effects of salinity and climate change on crop plants. *Scientia Horticulturae*, *78*(1-4), 159-174.

Shumaila, S. and Ullah, S. (2020). Mitigation of Salinity-Induced Damages in Capsicum Annum L. (Sweet Pepper) Seedlings Using Priming Techniques: A Future Perspective of Climate Change in the Region. *Communications in Soil Science and Plant Analysis*, *51*(12), 1602-1625.

Campbell, B. M., Vermeulen, S. J., Aggarwal, P. K., Corner-Dolloff, C., Girvetz, E., Loboguerrero, A. M. and Wollenberg, E. (2016). Reducing risks to food security from climate change. *Global Food Security*, *11*, 34-43.

Sofi, P. A., Rehman, K., Gull, M., Kumari, J., Djanaguiraman, M. and Prasad, P. V. V. (2021). Integrating root architecture and physiological approaches for improving drought tolerance in common bean (*Phaseolus vulgaris* L.). *Plant Physiology Reports*, *26*(1), 4-22.

Iqbal, M. S., Singh, A. K. and Ansari, M. I. (2020). Effect of drought stress on crop production. In *New frontiers in stress management for durable agriculture, Springer, Singapore*, 35-47.

Hasanuzzaman, M., Bhuyan, M. H. M., Zulfiqar, F., Raza, A., Mohsin, S. M., Mahmud, J. A. and Fotopoulos, V. (2020). Reactive oxygen species and antioxidant defense in plants under abiotic stress: Revisiting the crucial role of a universal defense regulator. *Antioxidants*, *9*(8), 681.

Munns, R. (2011). Plant adaptations to salt and water stress: differences and commonalities. *Advances in botanical research*, *57*, 1-32.

Gholamin, R. and Khayatnezhad, M. (2020). Assessment of the Correlation between Chlorophyll Content and Drought Resistance in Corn Cultivars (*Zea Mays*). *Helix-The Scientific Explorer| Peer Reviewed Bimonthly International Journal*, *10*(05), 93-97.

Hameed, A., Sheikh, M. A., Hameed, A., Farooq, T., Basra, S. M. A. and Jamil, A. (2014). Chitosan seed priming improves seed germination and seedling growth in wheat (*Triticum aestivum* L.) under osmotic stress induced by polyethylene glycol. *Philippine Agricultural Scientist*, *97*(3), 294-299.

Anonymous. (2003). Agriculture Statistics of Pakistan, Min. Food, Agric. Islamabad, Pakistan

Riaz, A., Younis, A., Taj, A. R., Karim, A., Tariq, U., Munir, S. and Riaz, S. (2013). Effect of drought stress on growth and flowering of marigold (*Tagetes erecta* L.). *Pakistan Journal of Botany*, *45*(S1), 123-131.

Qamar, R., Ghias, M., Hussain, F., Habib, S., Razzaq, M. K., Aslam, M. and Habib, I. (2018). Effect of drought on morpho-physiological traits of sunflower (*Helianthus annuus* L) hybrids and their parental inbred lines. *Pakistan Journal of Agricultural Research*, *31*(2).

Chandio, R. (2012). Thaar and other areas may face severe drought. The Nations. http://www.nation.com.pk/pakistan-newsnewspaper-daily-english-online/national/23-Aug-2012/tharother- areas-may-face-severe-drought-says-report (accessed 27-8-2021)

Akhtar, G., Rajwana, I. A., Farid, N., Younas, M., Sajjad, Y., Amin, M. and Ullah, S. (2019). 7. Effect of irrigation intervals on growth of annual flowers under climatic conditions of South Punjab, Pakistan. *Pure and Applied Biology (PAB)*, *8*(4), 2169-2177.

Anonymous. (2012). Asia, US plains facing water extraction crisis. The Express Turbine. http:// tribune.com.pk/story/419560/asia-us-plains-facing-water-extraction-crisis/ (assessed 11-8-21).

Yordanov, I., Velikova, V. and Tsonev, T. (2003). Plant responses to drought and stress tolerance. Bulg. J. Plant Physiol. *Special Issue*, 187-206.

Akhtar, D. S. (2010). Emerging challenges to Indus Waters Treaty. *Regional Studies*, *28*(4), 3-66.

Sharif, M., Jabbar, A., Niazi, M. A. and Mahr, A. B. (2016). Managing water availability and requirements in Pakistan: challenges and way forward. *J. Agric. Res*, *54*(1), 117-131.

Ashraf, M. (2012). Overview of drip irrigation: potential, issues and constraints. *Presentation in International Center for Agricultural Research in the Dry Areas (ICARDA), Pakistan on December*, *31*, 2012.

Farooq, M., Wahid, A., Kobayashi, N., Fujita, D. and Basra, S. M. A. (2009) Plant drought stress: effects, mechanisms and management. *Agronomy for Sustainable Development*, *29,* 185–212.

Chiappero, J., del Rosario Cappellari, L., Alderete, L. G. S., Palermo, T. B. and Banchio, E. (2019). Plant growth promoting rhizobacteria improve the antioxidant status in Mentha piperita grown under drought stress leading to an enhancement of plant growth and total phenolic content. *Industrial Crops and Products*, *139*, 111553.

Yamaguchi-Shinozaki, K. and Shinozaki, K. (2006). Transcriptional regulatory networks in cellular responses and tolerance to dehydration and cold stresses. *Annu. Rev. Plant Biol.*, *57*, 781-803.

Mohamed, H. I. and Abdel-Hamid, A. M. E. (2013). Molecular and biochemical studies for heat tolerance on four cotton genotypes. *Romanian Biotechnol Letters, 18*(6), 7223-7231.

El-Tayeb, M. A. (2006) Differential response of two *Vicia faba* cultivars to drought: growth, pigments, lipid peroxidation, organic solutes, catalase and peroxidase activity. *Acta Agron Hung, 54*, 25-37.

Farooq, M., Basra, S. M. A., Wahid, A., Ahmad, N. and Saleem, B. A. (2009). Improving the drought tolerance in rice (*Oryza sativa* L.) by exogenous application of salicylic acid. *Journal of Agronomy and Crop Science*, *195*(4), 237-246.

Hassan, M. U., Chattha, M. U., Khan, I., Chattha, M. B., Aamer, M., Nawaz, M. and Khan, T. A. (2019). Nickel toxicity in plants: reasons, toxic effects, tolerance mechanisms, and remediation possibilities—a review. *Environmental Science and Pollution Research*, *26*(13), 12673-12688.

Kachout, S. S., Hamza, K. J., Bouraoui, N. K., Leclerc, J. C. and Ouerghi, Z. (2013). Salt-induced changes in antioxidative enzyme activities in shoot tissues of two Atriplex varieties. *Notulae Botanicae Horti Agrobotanici Cluj-Napoca*, *41*(1), 115-121.

Yang, R., Howe, J. A. and Golden, B. R. (2019). Calcium silicate slag reduces drought stress in rice (*Oryza sativa* L.). *Journal of Agronomy and Crop Science*, *205*(4), 353-361.

Gammoudi, N., Yahia, L. B., Lachiheb, B. and Ferchichi, A. (2016). Salt response in pepper (*Capsicum annuum* L.): components of photosynthesis inhibition, proline accumulation and K+/Na+ selectivity. *Journal of Aridland Agriculture*.

Beddington, J. R., Asaduzzaman, M., Clark, M. E., Bremauntz, A. F., Guillou, M. D., Howlett, D. J. B. and Wakhungu, J. (2012). What next for agriculture after Durban? *Science*, *335*(6066), 289-290.

Wang, W., Wu, Z., He, Y., Huang, Y., Li, X. and Ye, B. C. (2018). Plant growth promotion and alleviation of salinity stress in *Capsicum annuum* L. by Bacillus isolated from saline soil in Xinjiang. *Ecotoxicology and Environmental Safety*, *164*, 520-529.

Karimi, M., Ahmadi, A., Hashemi, J., Abbasi, A., Tavarini, S., Pompeiano, A. and Angelini, L. G. (2019). Plant growth retardants (PGRs) affect growth and secondary metabolite biosynthesis in Stevia rebaudiana Bertoni under drought stress. *South African Journal of Botany*, *121*, 394-401.

Hu, X., Xu, Z., Xu, W., Li, J., Zhao, N. and Zhou, Y. (2015). Application of γ-aminobutyric acid demonstrates a protective role of polyamine and GABA metabolism in muskmelon seedlings under Ca (NO3) 2 stress. *Plant Physiology and Biochemistry*, *92*, 1-10.

Jin, X., Liu, T., Xu, J., Gao, Z. and Hu, X. (2019). Exogenous GABA enhances muskmelon tolerance to salinity-alkalinity stress by regulating redox balance and chlorophyll biosynthesis. *BMC plant biology*, *19*(1), 1-15.

Narsai, R., Rocha, M., Geigenberger, P., Whelan, J. and van Dongen, J. T. (2011). Comparative analysis between plant species of transcriptional and metabolic responses to hypoxia. *New Phytologist*, *190*(2), 472-487.

Signorelli, S., Dans, P. D., Coitiño, E. L., Borsani, O. and Monza, J. (2015). Connecting proline and γ-aminobutyric acid in stressed plants through non-enzymatic reactions. *PLoS One*, *10*(3), 115-349.

Salah, A., Zhan, M., Cao, C., Han, Y., Ling, L., Liu, Z. and Jiang, Y. (2019). γ-Aminobutyric acid promotes chloroplast ultrastructure, antioxidant capacity, and growth of waterlogged maize seedlings. *Scientific reports*, *9*(1), 1-19.

Guo, M., Liu, J. H., Ma, X., Zhai, Y. F., Gong, Z. H. and Lu, M. H. (2016) Genome-wide analysis of the Hsp70 family genes in pepper (*Capsicum annuum* L.) and functional identification of CaHsp70-2 involvement in heat stress. *Plant Science*, *252*, 246-256.

Shumaila, S. and Ullah, S. (2020). Mitigation of Salinity-Induced Damages in Capsicum Annum L. (Sweet Pepper) Seedlings Using Priming Techniques: A Future Perspective of Climate Change in the Region. *Communications in Soil Science and Plant Analysis*, *51*(12), 1602-1625.

Kumar, S., Kumar, R. and Singh, J. (2006). Cayenne/American pepper. In *Handbook of herbs and spices*. *Woodhead Publishing*, 299-312.

Naz, S., Anjum, M. A. and Ahmad, I. (2006). Growth of Chilli (*Capsicum annuum* L.) F1 hybrid sky line-2 in response to different ages of transplants. *J Res (Sci)*, *17*, 91-95.

Kumar, M., Singh, R. P. and Bose, B. (2018). Effect of seed priming on the activity of antioxidant enzymes and changes of biochemicals of rice under timely and late sown conditions. *International Journal of Current Microbiology and Applied Sciences, 7*(5), 2985-92.

Khan, S., Khan, M. A., Hanjra, M. A. and Mu, J. (2009). Pathways to reduce the environmental footprints of water and energy inputs in food production. *Food policy*, *34*(2), 141-149.

Nadeem, M., Muhammad, F., Anjum, M., Khan, R., Saeed, M. and Riaz, A. (2014). National Institute of Food Science and Technology, University of Agriculture, Faisalabad-Pakistan.

Bibi, Z., Khan, N. U., Latif, A., Khan, M. J., Manyuan, G., Niu, Y. and Sadozai, G. U. (2016). Salt tolerance in upland cotton genotypes to NaCl salinity at early growth stages. *JAPS: Journal of Animal and Plant Sciences*, *26*(3).

Khan, S., Khan, M. A., Hanjra, M. A. and Mu, J. (2009). Pathways to reduce the environmental footprints of water and energy inputs in food production. *Food policy*, *34*(2), 141-149.

Thomas, R. R., Prakash, M. C., Reddy, M. K., Mahmood, R. and Mondal, P. (2017). Identification of dehydrin markers in *Capsicum annuum* for development of drought tolerant crops in changing environment.

Campos H., Trejo C., Pen˜a-Valdivia C. B., Garcı´a-Nava R., CondeMartı´nez F. V. and Cruz-Ortega M. R. (2014) Stomatal and nonstomatal limitations of bell pepper (*Capsicum annuum* L.) plants under water stress and re-watering: delayed restoration of photosynthesis during recovery. *Environ Exp, 98*, 56–64.

McLean, E. O. (1983). Soil pH and lime requirement. *Methods of soil analysis: Part 2 Chemical and microbiological properties*, *9*, 199-224.

Nelson, D. W. and Sommers, L. E. (1982). Total carbon, organic carbon and organic matter: In: Page AL, Miller RH, Keeney DR (eds) Methods of soil analysis. *Part*, *2*, 539-579.

Keeney, D. R. and Nelson, D. W. (1982). Nitrogen-inorganic forms in Methods of Soil Analysis, Part 2. Chemical and Microbiological Properties, SSSA Book Series No 9. *Soil Science Society of America and American Society of Agronomy, Madison, WI*, 643-698.

Jackson, M. L. (1973). Vanadomolybdo phosphoric yellow colour method for determination of phosphorus. *Soil Chemical Analysis*, 151-154.

Hanway, J. J. and Heidel, H. (1952). Soil analyses methods as used in Iowa state college soil testing laboratory. *Iowa Agriculture, 57*, 1–31.

Bouyoucos, G. J. (1962). Hydrometer method improved for making particle size analyses of soils 1. *Agro. J., 54*(5), 464-465.

Ullah, S., Zada, J. and Ali, S. (2016). Effect of nephthyl acetic acid foliar spray on amelioration of drought stress tolerance in maize (Zea mays L.). *Communications in Soil Science and Plant Analysis*, *47*(12), 1542-1558.

Hussain, J., Rehman, N., Khan, A. L., Hussain, H., Al-Harrasi, A., Ali, L. and Shinwari, Z. K. (2011). Determination of macro and micronutrients and nutritional prospects of six vegetable species of Mardan, Pakistan. *Pak. J. Bot*, *43*(6), 2829-2833.

Ahmed, A. F., Yu, H., Yang, X. and Jiang, W. (2014). Deficit irrigation affects growth, yield, vitamin C content, and irrigation water use efficiency of hot pepper grown in soilless culture. *HortScience, 49*(6), 722-728.

Chuyong, G. B. and Acidri, T. (2017). Light and moisture levels affect growth and physiological parameters differently in *Faidherbia albida* (Delile) A. Chev. Seedlings. *Acta Physiologiae Plantarum*, *39*(5), 1-6.

Ali, Q. and Ashraf, M. (2011). Induction of drought tolerance in maize (*Zea mays* L.) due to exogenous application of trehalose: growth, photosynthesis, water relations and oxidative defence mechanism. *Journal of Agronomy and Crop Science*, *197*(4), 258-271.

Barranco, D., Ruiz, N. and Gómez-del Campo, M. (2005). Frost tolerance of eight olive cultivars. *Hort Science*, *40*(3), 558-560.

Zou, M., Yuan, L., Zhu, S., Liu, S., Ge, J. and Wang, C. (2017). Effects of heat stress on photosynthetic characteristics and chloroplast ultrastructure of a heat-sensitive and heat-tolerant cultivar of wucai (*Brassica campestris* L.). *Acta physiologiae plantarum*, *39*(1), 1-10.

Marcińska, I., Czyczyło-Mysza, I., Skrzypek, E., Grzesiak, M. T., Popielarska-Konieczna, M., Warchoł, M. and Grzesiak, S. (2017). Application of photochemical parameters and several indices based on phenotypical traits to assess intraspecific variation of oat (*Avena sativa* L.) tolerance to drought. *Acta Physiologiae Plantarum*, *39*(7), 1-13.

Płażek, A., Tatrzańska, M., Maciejewski, M., Kościelniak, J., Gondek, K., Bojarczuk, J. and Dubert, F. (2013). Investigation of the salt tolerance of new Polish bread and durum wheat cultivars. *Acta Physiologiae Plantarum*, *35*(8), 2513-2523.

Lowery, O. A., Poesenbrough, N. J., Fal, A. L. and Randall, R. J. (1951). Protein measurement with foline phenol reagent. *The Journal of Biological Chemistry, 193*, 265–75.

Asthir, B., Kaur, S. and Mann, S. K. (2009). Effect of salicylic and abscisic acid administered through detached tillers on antioxidant system in developing wheat grains under heat stress. *Acta physiologiae plantarum, 31*(5), 1091-1096.

1107.

Yuan, Y., Qian, H., Yu, Y., Lian, F. and Tang, D. (2011). Thermo tolerance and antioxidant response induced by heat acclimation in Freesia seedlings. *Acta Physiologiae Plantarum*, *33*(3), 1001-1009.

Tyburski, J., Dunajska, K., Mazurek, P., Piotrowska, B. and Tretyn, A. (2009). Exogenous auxin regulates H_2O_2 metabolism in roots of tomato (*Lycopersicon esculentum Mill*.) seedlings affecting the expression and activity of CuZn-superoxide dismutase, catalase, and peroxidase. *Acta physiologiae plantarum*, *31*(2), 249.

Salimi, F., Shekari, F. and Hamzei, J. (2016). Methyl jasmonate improves salinity resistance in German chamomile (*Matricaria chamomilla* L.) by increasing activity of antioxidant enzymes. *Acta physiologiae plantarum*, *38*(1), 1

Kosar, F., Akram, N. A. and Ashraf, M. (2015). Exogenously-applied 5-aminolevulinic acid modulates some key physiological characteristics and antioxidative defense system in spring wheat (*Triticum aestivum* L.) seedlings under water stress. *South African Journal of Botany*, *96*, 71-77.

Tesfamariam, E. H., Annandale, J. G. and Steyn, J. M. (2010). Water stress effects on winter canola growth and yield. *Agronomy Journal*, *102*(2), 658-666.

Tripathy, B. C. and Oelmüller, R. (2012). Reactive oxygen species generation and signaling in plants. *Plant signaling and behavior*, *7*(12), 1621-1633.

Dolatabadian, A., Modarres Sanavy, S. A. M. and Sharifi, M. (2009). Alleviation of water deficit stress effects by foliar application of ascorbic acid on Zea mays L. *Journal of Agronomy and Crop Science*, *195*(5), 347-355.

Singh, A. and Roychoudhury, A. (2020). Role of γ-Aminobutyric Acid in the Mitigation of Abiotic Stress in Plants. *Protective Chemical Agents in the Amelioration of Plant Abiotic Stress: Biochemical and Molecular Perspectives*, 413-423.

Bashir, R., Riaz, H. N., Anwar, S., Parveen, N., Khalilzadeh, R., Hussain, I. and Mahmood, S. (2021). Morpho-physiological changes in carrots by foliar γ-aminobutyric acid under drought stress. *Brazilian Journal of Botany*, *44*(1), 57-68.

Vijayakumari, K., Jisha, K. C. and Puthur, J. T. (2016). GABA/BABA priming: a means for enhancing abiotic stress tolerance potential of plants with less energy investments on defence cache. *Acta Physiologiae Plantarum*, *38*(9), 1-14.

Nayyar, H., Kaur, R., Kaur, S. and Singh, R. (2014). γ-Aminobutyric acid (GABA) imparts partial protection from heat stress injury to rice seedlings by improving leaf turgor and upregulating osmoprotectants and antioxidants. *Journal of Plant Growth Regulation*, *33*(2), 408-419.

Li, Z., Yu, J., Peng, Y. and Huang, B. (2016). Metabolic pathways regulated by γ-aminobutyric acid (GABA) contributing to heat tolerance in creeping bentgrass (*Agrostis stolonifera*). *Scientific Reports*, *6*(1), 1-16.

Wang, Y., Luo, Z., Mao, L. and Ying, T. (2016). Contribution of polyamines metabolism and GABA shunt to chilling tolerance induced by nitric oxide in cold-stored banana fruit. *Food chemistry*, *197*, 333-339.

Malekzadeh, P., Khara, J. and Heydari, R. (2014). Alleviating effects of exogenous Gamma-aminobutiric acid on tomato seedling under chilling stress. *Physiology and Molecular Biology of Plants*, *20*(1), 133-137.

Palma, F., Carvajal, F., Jiménez-Muñoz, R., Pulido, A., Jamilena, M. and Garrido, D. (2019). Exogenous γ-aminobutyric acid treatment improves the cold tolerance of zucchini fruit during postharvest storage. *Plant physiology and biochemistry*, *136*, 188-195.

Malekzadeh, P., Khosravi-Nejad, F., Hatamnia, A. A. and Mehr, R. S. (2017). Impact of postharvest exogenous γ-aminobutyric acid treatment on cucumber fruit in response to chilling tolerance. *Physiology and Molecular Biology of Plants*, *23*(4), 827-836.

Bustamante, C. A., Monti, L. L., Gabilondo, J., Scossa, F., Valentini, G., Budde, C. O. and Drincovich, M. F. (2016). Differential metabolic rearrangements after cold storage are correlated with chilling injury resistance of peach fruits. *Frontiers in plant science*, *7*, 1478.

Abdel Razik, E. S., Alharbi, B. M., Pirzadah, T. B., Alnusairi, G. S., Soliman, M. H. and Hakeem, K. R. (2021). γ-Aminobutyric acid (GABA) mitigates drought and heat stress in sunflower (Helianthus annuus L.) by regulating its physiological, biochemical and molecular pathways. *Physiologia Plantarum*, *172*(2), 505-527.

Shafiq, S., Akram, N. A., Ashraf, M. and Arshad, A. (2014). Synergistic effects of drought and ascorbic acid on growth, mineral nutrients and oxidative defense system in canola (*Brassica napus* L.) plants. *Acta Physiologiae Plantarum*, *36*(6), 1539-1553.

Furlan, A., Llanes, A., Luna, V. and Castro, S. (2013). Abscisic acid mediates hydrogen peroxide production in peanut induced by water stress. *Biologia plantarum*, *57*(3), 555-558.

Ma, X., Zhu, C., Yang, N., Gan, L. and Xia, K. (2016). γ-Aminobutyric acid addition alleviates ammonium toxicity by limiting ammonium accumulation in rice (*Oryza sativa*) seedlings. *Physiologia Plantarum*, *158*(4), 389-401.

Cheng, B., Li, Z., Liang, L., Cao, Y., Zeng, W., Zhang, X. and Peng, Y. (2018). The γ-aminobutyric acid (GABA) alleviates salt stress damage during seeds germination of white clover associated with Na+/K+ transportation, dehydrins accumulation, and stress-related genes expression in white clover. *International Journal of Molecular Sciences*, *19*(9), 2520.

Mahmud, J. A., Hasanuzzaman, M., Nahar, K., Rahman, A., Hossain, M. and Fujita, M. (2017). γ-aminobutyric acid (GABA) confers chromium stress tolerance in *Brassica juncea* L. by modulating the antioxidant defense and glyoxalase systems. *Ecotoxicology*, *26*(5), 675-690.

Soltys-Kalina, D., Plich, J., Strzelczyk-Żyta, D., Śliwka, J. and Marczewski, W. (2016). The effect of drought stress on the leaf relative water content and tuber yield of a half-sib family of 'Katahdin'-derived potato cultivars. *Breeding science*, *66*(2), 328-331.

Lukatkin, A. S. and Anjum, N. A. (2014). Control of cucumber (*Cucumis sativus* L.) tolerance to chilling stress—evaluating the role of ascorbic acid and glutathione. *Frontiers in Environmental Science*, *2*, 62.

Hemmati, K., Ebadi, A., Khomari, S. and Sedghi, M. (2018). Influence of ascorbic acid and 24-epibrassinolide on physiological characteristics of pot marigold under water-stress condition. *Journal of Plant Interactions*, *13*(1), 364-372.

Yong, B., Xie, H., Li, Z., Li, Y. P., Zhang, Y., Nie, G. and Peng, Y. (2017). Exogenous application of GABA improves PEG-induced drought tolerance positively associated with GABA-shunt, polyamines, and proline metabolism in white clover. *Frontiers in physiology*, *8*, 1107.

Wang, X., Xie, B., An, W., Li, J., Zhai, Z. and Duan, L. (2009). Effects of exogenous GABA on yield, quality and high temperature tolerance of winter wheat at the anthesis stage. *Journal of Triticeae Crops*, *29*(4), 623-626.

Chéour, F., Kaddachi, I., Achouri, D., Bannour, S. and Zorgui, L. (2014). Effects of water stress on relative water, chlorophylls and proline contents in barley (*Hordeum vulgare* L.) leaves. *IOSR J. Agric. Vet. Sci. IOSR-JAVS*, *7*, 13-16.

Ebrahimian, E. and Bybordi, A. (2012). Influence of ascorbic acid foliar application on chlorophyll, flavonoids, anthocyanin and soluble sugar contents of sunflower under conditions of water deficit stress. *Journal of food, Agriculture and Environment*, *10*(1 part 2), 1026-1030.

Ebrahimian, E. and Bybordi, A. (2011). Influence of different ratios of nitrate and ammonium and silicon on growth, nitrate reductase activity and fatty acid composition of sunflower under salt stress. *Journal of Food, Agriculture and Environment*, *9*(1), 438-443.

Rezaei-Chiyaneh, E., Seyyedi, S. M., Ebrahimian, E., Moghaddam, S. S. and Damalas, C. A. (2018). Exogenous application of gamma-aminobutyric acid (GABA) alleviates the effect of water deficit stress in black cumin (*Nigella sativa* L.). *Industrial Crops and Products*, *112*, 741-748.

Liu, B., Liang, J., Tang, G., Wang, X., Liu, F. and Zhao, D. (2019). Drought stress affects on growth, water use efficiency, gas exchange and chlorophyll fluorescence of Juglans rootstocks. *Scientia horticulturae*, *250*, 230-235

Li, H., Mo, Y., Cui, Q., Yang, X., Guo, Y., Wei, C. and Zhang, X. (2019). Transcriptomic and physiological analyses reveal drought adaptation strategies in drought-tolerant and-susceptible watermelon genotypes. *Plant Science*, *278*, 32-43.

Jisha, K. C. and Puthur, J. T. (2016). Seed priming with BABA (β-amino butyric acid): a cost-effective method of abiotic stress tolerance in *Vigna radiata* (L.) Wilczek. *Protoplasma*, *253*(2), 277-289.

Wattanakulpakin, P., Photchanachai, S., Ratanakhanokchai, K., Kyu, K. L., Ritthichai, P. and Miyagawa, S. (2012). Hydropriming effects on carbohydrate metabolism, antioxidant enzyme activity and seed vigor of maize (Zea mays L.). *African Journal of Biotechnology*, *11*(15), 3537-3547.

Hayat, S., Hayat, Q., Alyemeni, M. N., Wani, A. S., Pichtel, J. and Ahmad, A. (2012). Role of proline under changing environments: a review. *Plant signaling and behavior*, *7*(11), 1456-1466.

Pirzadah, T. B., Malik, B., Tahir, I., Rehman, R. U., Hakeem, K. R. and Alharby, H. F. (2019). Aluminium stress modulates the osmolytes and enzyme defense system in Fagopyrum species. *Plant Physiology and Biochemistry*, *144*, 178-186.

Ahanger, M. A., Tomar, N. S., Tittal, M., Argal, S. and Agarwal, R. M. (2017). Plant growth under water/salt stress: ROS production; antioxidants and significance of added potassium under such conditions. *Physiology and Molecular Biology of Plants*, *23*(4), 731-744.

Soliman, M., Alhaithloul, H. A., Hakeem, K. R., Alharbi, B. M., El-Esawi, M. and Elkelish, A. (2019). Exogenous nitric oxide mitigates nickel-induced oxidative damage in eggplant by upregulating antioxidants, osmolyte metabolism, and glyoxalase systems. *Plants*, *8*(12), 562.

Seifikalhor, M., Aliniaeifard, S., Bernard, F., Seif, M., Latifi, M., Hassani, B. and Li, T. (2020). γ-Aminobutyric acid confers cadmium tolerance in maize plants by concerted regulation of polyamine metabolism and antioxidant defense systems. *Scientific reports*, *10*(1), 1-18.

Yong, B., Xie, H., Li, Z., Li, Y. P., Zhang, Y., Nie, G. and Peng, Y. (2017). Exogenous application of GABA improves PEG-induced drought tolerance positively associated with GABA-shunt, polyamines, and proline metabolism in white clover. *Frontiers in physiology*, *8*, 1107.

Barbosa, J. M., Singh, N. K., Cherry, J. H. and Locy, R. D. (2010). Nitrate uptake and utilization is modulated by exogenous γ-aminobutyric acid in Arabidopsis thaliana seedlings. *Plant Physiology and Biochemistry*, *48*(6), 443-450.

Babaee, K., Amini Dehaghi, M., Modares Sanavi, S. A. M. and Jabbari, R. (2010). Water deficit effect on morphology, prolin content and thymol percentage of Thyme (*Thymus vulgaris* L.). *Iranian Journal of Medicinal and Aromatic Plants Research*, *26*(2), 239-251.

Krishnan, S., Laskowski, K., Shukla, V. and Merewitz, E. B. (2013). Mitigation of drought stress damage by exogenous application of a non-protein amino acid γ–aminobutyric acid on perennial ryegrass. *Journal of the American Society for Horticultural Science*, *138*(5), 358-366.

Laxa, M., Liebthal, M., Telman, W., Chibani, K. and Dietz, K. J. (2019). The role of the plant antioxidant system in drought tolerance. *Antioxidants*, *8*(4), 94.

Balouchi, H. and Ahmadpour Dehkordi, S. (2013). Effect of different seed priming on germination traits in Black cumin (*Nigella sativa*) under salinity stress. *Journal of Plant Production Research*, *20*(3), 1-26.

PONE-D-23-13341.pdf

ORIGINALITY REPORT

37%
SIMILARITY INDEX

PRIMARY SOURCES

1	Shumaila Shumaila, Sami Ullah. " Mitigation of Salinity-Induced Damages in L. (Sweet Pepper) Seedlings Using Priming Techniques: A Future Perspective of Climate Change in the Region ", Communications in Soil Science and Plant Analysis, 2020<br>Crossref	1104 words — 7%
2	www.medrxiv.org<br>Internet	475 words — 3%
3	link.springer.com<br>Internet	372 words — 2%
4	Hany G. Abd El-Gawad, Soumya Mukherjee, Reham Farag, Ola H. Abd Elbar et al. " Exogenous γ-aminobutyric acid (GABA)-induced signaling events and field performance associated with mitigation of drought stress in L ", Plant Signaling & Behavior, 2020<br>Crossref	357 words — 2%
5	www.biorxiv.org<br>Internet	281 words — 2%
6	www.researchgate.net<br>Internet	214 words — 1%
7	Sami Ullah, Muhammad Ishaq Khan, Muhammad Nauman Khan, Usman Ali et al. " Efficacy of	205 words — 1%

Naphthyl Acetic Acid Foliar Spray in Moderating Drought Effects on the Morphological and Physiological Traits of Maize Plants (L.) ", ACS Omega, 2023
Crossref

8	www.research.ed.ac.uk<br>Internet	204 words — 1%
9	SAMI ULLAH, ISHFAQ AFZAL, SHUM AILA, WADOOD SHAH. "nnnEffect Of Naphthyl Acetic Acid Foliar Spray On The Physiological Mechanism Of Drought Stress Tolerance In Maize (Zea Mays L.)", Plant Stress, 2021<br>Crossref	171 words — 1%
10	journalarticle.ukm.my<br>Internet	98 words — 1%
11	www.preprints.org<br>Internet	98 words — 1%
12	boris.unibe.ch<br>Internet	82 words — 1%
13	Jungin Lee, Olivia J. Cook, Andrea P. Argüelles, Yashar Mehmani. "Imaging geomechanical properties of shales with infrared light", Fuel, 2023<br>Crossref	79 words — 1%
14	Eltohamy A. A. Yousef Ibrahim N. Nasef. "Growth and Yield Response of Garlic Genotypes to Foliar Application of γ-Aminobutyric Acid", Hortscience Journal of Suez Canal University, 2019<br>Crossref	78 words — 1%
15	Esmaeil Rezaei-Chiyaneh, Seyyed Mohammad Seyyedi, Elnaz Ebrahimian, Sina Siavash Moghaddam, Christos A. Damalas. "Exogenous application of	74 words — < 1%

gamma-aminobutyric acid (GABA) alleviates the effect of water deficit stress in black cumin (Nigella sativa L.)", Industrial Crops and Products, 2018
Crossref

16	storage.googleapis.com<br>Internet	71 words — < 1%
17	Nazish Raees, Sami Ullah, Muhammad Nafees. "Interactive Effect of Tocopherol, Salicylic Acid and Ascorbic Acid on Agronomic Characters of Two Genotypes of Brassica napusL. Under Induced Drought and Salinity Stresses", Gesunde Pflanzen, 2022<br>Crossref	68 words — < 1%
18	www.frontiersin.org<br>Internet	53 words — < 1%
19	www.mdpi.com<br>Internet	51 words — < 1%
20	facultystaff.urmia.ac.ir<br>Internet	45 words — < 1%
21	helda.helsinki.fi<br>Internet	41 words — < 1%
22	theses.hal.science<br>Internet	39 words — < 1%
23	mdpi-res.com<br>Internet	38 words — < 1%
24	aloki.hu<br>Internet	36 words — < 1%
25	repository.uma.ac.ir<br>Internet	

36 words — < 1%

26	www.sphinxsai.com<br>Internet	34 words — < 1%
27	"Water Stress and Crop Plants", Wiley, 2016<br>Crossref	32 words — < 1%
28	eprints.utas.edu.au<br>Internet	30 words — < 1%
29	dokumen.pub<br>Internet	29 words — < 1%
30	journals.plos.org<br>Internet	29 words — < 1%
31	Shumaila, Sami Ullah, Muhammad Nafees. "Biochar Application to Soil and Seed Pre-Soaking on Growth, Yield and Physiological Response of Solanum melongena L. Under Induced Abiotic Stresses", Journal of Plant Growth Regulation, 2023<br>Crossref	28 words — < 1%
32	Zahra Khazaei, Asghar Estaji. "Effect of foliar application of ascorbic acid on sweet pepper (Capsicum annuum) plants under drought stress", Acta Physiologiae Plantarum, 2020<br>Crossref	28 words — < 1%
33	ijrbat.in<br>Internet	26 words — < 1%
34	www.repository.cam.ac.uk<br>Internet	26 words — < 1%

35	www.tandfonline.com<br>Internet	26 words — < 1%
36	Raghad S. Mouhamad, Munawar Iqbal, M. Afzal Qamar, Lamiaa A. Mutlag, Ibrahim B. Razaq, Mazhar Abbas, Fida Hussain. "Effect of gravistimulation on amino acid profile of pea, rice, corn, wheat during early growth stages", Information Processing in Agriculture, 2016<br>Crossref	24 words — < 1%
37	hdl.handle.net<br>Internet	23 words — < 1%
38	mafiadoc.com<br>Internet	23 words — < 1%
39	journals.uni-lj.si<br>Internet	22 words — < 1%
40	Gul Lalay, Sami Ullah, Iftikhar Ahmed. " Physiological and biochemical responses of L. to stress by the application of biochar and ", Microscopy Research and Technique, 2021<br>Crossref	21 words — < 1%
41	Ojasvini Ahluwalia, Poonam C. Singh, Ranjana Bhatia. "A review on drought stress in plants: Implications, mitigation and the role of plant growth promoting rhizobacteria", Resources, Environment and Sustainability, 2021<br>Crossref	20 words — < 1%
42	iksadyayinevi.com<br>Internet	19 words — < 1%
43	"Protective Chemical Agents in the Amelioration of Plant Abiotic Stress", Wiley, 2020<br>Crossref	18 words — < 1%

44 Mobeen Ahmed Khan, Muhammad Asif Asghar, Javed Iqbal, Aftab Ahmed, Zuzzer Ali Shamsuddin. " Aflatoxins contamination and prevention in red chillies ( ) in Pakistan ", Food Additives & Contaminants: Part B, 2013 — 18 words — < 1%
Crossref

45 vdoc.pub — 17 words — < 1%
Internet

46 www2.mdpi.com — 17 words — < 1%
Internet

47 "Microbial Probiotics for Agricultural Systems", Springer Science and Business Media LLC, 2019 — 16 words — < 1%
Crossref

48 Wiem Mnafgui, Hichem Hajlaoui, Valeria Rizzo, Giuseppe Muratore, Amine Elleuch. "Priming with EDTA, IAA and Fe Alleviates Pb Toxicity in Trigonella Foneum graecum L. growth: Phytochemicals and secondary metabolites", Journal of Biotechnology, 2022 — 16 words — < 1%
Crossref

49 archive.conscientiabeam.com — 16 words — < 1%
Internet

50 researchrepository.murdoch.edu.au — 16 words — < 1%
Internet

51 Khyrollah Babaei, Mohammad Moghaddam, Nasrin Farhadi, Abdollah Ghasemi Pirbalouti. "Morphological, physiological and phytochemical responses of Mexican marigold (Tagetes minuta L.) to drought stress", Scientia Horticulturae, 2021 — 14 words — < 1%
Crossref

52 Noor Ali Shah, Sami Ullah, Muhammad Nafees, Muhammad Nauman Khan. "Exogenous Effect of Sugar Beet Extract On Physio-biochemical Traits of Hordeum vulagreL. Under Induced Salinity Stress", Gesunde Pflanzen, 2023 — 14 words — < 1%
Crossref

53 intl.plantphysiol.org — 13 words — < 1%
Internet

54 Xuedong Wang, Yanfeng Sun, Shiyu Li, Hanxi Wang. "Spatial distribution and ecological risk assessment of heavy metals in soil from the Raoyanghe Wetland, China", PLOS ONE, 2019 — 12 words — < 1%
Crossref

55 Zhou Li, Yan Peng, Bingru Huang. "Alteration of Transcripts of Stress-Protective Genes and Transcriptional Factors by γ-Aminobutyric Acid (GABA) Associated with Improved Heat and Drought Tolerance in Creeping Bentgrass (Agrostis stolonifera)", International Journal of Molecular Sciences, 2018 — 12 words — < 1%
Crossref

56 "Microbial BioTechnology for Sustainable Agriculture Volume 1", Springer Science and Business Media LLC, 2022 — 11 words — < 1%
Crossref

57 Liu, Hui, Chuying Yu, Hanxia Li, Bo Ouyang, Taotao Wang, Junhong Zhang, Xin Wang, and Zhibiao Ye. "Overexpression of ShDHN, a dehydrin gene from Solanum habrochaites enhances tolerance to multiple abiotic stresses in tomato", Plant Science, 2015. — 11 words — < 1%
Crossref

58 Sumaiya Farzana, Md. Rasel, Md. Tahjib Ul Arif, Mohammad Anwar Hossain et al. "Salicylic acid and thiourea ameliorate the negative impact of salt stress in wheat (Triticum aestivum L.) seedlings by up-regulating photosynthetic pigments, leaf water status, and antioxidant defense system", Journal of Phytology, 2021
Crossref
11 words — < 1%

59 researchonline.jcu.edu.au
Internet
11 words — < 1%

60 www.emerald.com
Internet
11 words — < 1%

61 www.spkx.net.cn
Internet
11 words — < 1%

62 "Abiotic Stress Response in Plants", Wiley, 2016
Crossref
10 words — < 1%

63 Maryam Yousefzadeh Najafabadi, Parviz Ehsanzadeh. "Salicylic Acid Effects on Osmoregulation and Seed Yield in Drought-Stressed Sesame", Agronomy Journal, 2017
Crossref
10 words — < 1%

64 Sami Ullah, Sara Ali, Afroz Binte Abid, Muhammad Nafees. " Modulating response of to induced salinity stress through application of nitrate mediated silver nanoparticles and indole acetic acid ", Microscopy Research and Technique, 2021
Crossref
10 words — < 1%

65 docsdrive.com
Internet
10 words — < 1%

etheses.whiterose.ac.uk

66 Internet 10 words — < 1%

67 journalofscience.org
Internet
10 words — < 1%

68 journals.ashs.org
Internet
10 words — < 1%

69 pakbs.org
Internet
10 words — < 1%

70 trace.tennessee.edu
Internet
10 words — < 1%

71 updatepublishing.com
Internet
10 words — < 1%

72 www.joasdjournal.com
Internet
10 words — < 1%

73 "Managing Plant Production Under Changing Environment", Springer Science and Business Media LLC, 2022
Crossref
9 words — < 1%

74 Eszter Nemeskéri, Éva Sárdi, Judit Remenyik, Béla Kőszegi, Pál Nagy. "Study of the defensive mechanism against drought in French bean (Phaseolus vulgaris L.) varieties", Acta Physiologiae Plantarum, 2010
Crossref
9 words — < 1%

75 Fang-Lan He, Ai-Ke Bao, Suo-Min Wang, Hong-Xi Jin. "NaCl stimulates growth and alleviates drought stress in the salt-secreting xerophyte Reaumuria soongorica", Environmental and Experimental Botany, 2019
Crossref
9 words — < 1%

76 Mohammad Hossein Sheikh Mohammadi, Nematollah Etemadi, Mohammad Mehdi Arab, Mostafa Aalifar, Mostafa Arab, Mohammad Pessarakli. "Molecular and physiological responses of Iranian Perennial ryegrass as affected by Trinexapac ethyl, Paclobutrazol and Abscisic acid under drought stress", Plant Physiology and Biochemistry, 2017 — 9 words — < 1%
Crossref

77 Muhammad Nafees, Sami Ullah, Iftikhar Ahmed. " Morphological and elemental evaluation of biochar through analytical techniques and its combined effect along with plant growth promoting rhizobacteria on L. under induced drought stress ", Microscopy Research and Technique, 2021 — 9 words — < 1%
Crossref

78 Wild Edible Vegetables of Lesser Himalayas, 2015. — 9 words — < 1%
Crossref

79 downloads.hindawi.com — 9 words — < 1%
Internet

80 eprints.nottingham.ac.uk — 9 words — < 1%
Internet

81 ir.canterbury.ac.nz — 9 words — < 1%
Internet

82 ses.library.usyd.edu.au — 9 words — < 1%
Internet

83 www.cclean.org — 9 words — < 1%
Internet

84 www.mbao.org — 9 words — < 1%
Internet

85 www.ncbi.nlm.nih.gov
Internet
9 words — < 1%

86 www.sciencegate.app
Internet
9 words — < 1%

87 www.ukaazpublications.com
Internet
9 words — < 1%

88 "Cotton Production and Uses", Springer Science and Business Media LLC, 2020
Crossref
8 words — < 1%

89 "Sustainable Agriculture Reviews 53", Springer Science and Business Media LLC, 2021
Crossref
8 words — < 1%

90 Arezoo Paravar, Saeideh Maleki Farahani, Alireza Rezazadeh. "Lallemantia species response to drought stress and Arbuscular mycorrhizal fungi application", Industrial Crops and Products, 2021
Crossref
8 words — < 1%

91 Bashri, Gausiya, and Sheo Mohan Prasad. "Exogenous IAA differentially affects growth, oxidative stress and antioxidants system in Cd stressed Trigonella foenum-graecum L. seedlings: Toxicity alleviation by up-regulation of ascorbate-glutathione cycle", Ecotoxicology and Environmental Safety, 2016.
Crossref
8 words — < 1%

92 C.K. Singh, B.K. Rajkumar, V. Kumar. "Differential responses of antioxidants and osmolytes in upland cotton (Gossypium hirsutum) cultivars contrasting in drought tolerance", Plant Stress, 2021
Crossref
8 words — < 1%

93 El-Sayed M. Desoky, Ahmed M. Saad, Mohamed T. El-Saadony, Abdel-Rahman M. Merwad, Mostafa M. Rady. "Plant growth-promoting rhizobacteria: Potential improvement in antioxidant defense system and suppression of oxidative stress for alleviating salinity stress in Triticum aestivum (L.) plants", Biocatalysis and Agricultural Biotechnology, 2020
Crossref

8 words — < 1%

94 Ibrahim, Mohd Hafiz, Hawa Z.E. Jaafar, Asmah Rahmat, and Zaharah Abdul Rahman. "Effects of Nitrogen Fertilization on Synthesis of Primary and Secondary Metabolites in Three Varieties of Kacip Fatimah (Labisia Pumila Blume)", International Journal of Molecular Sciences, 2011.
Crossref

8 words — < 1%

95 Nihal El Nahhas, Muneera D.F. AlKahtani, Khaled A.A. Abdelaal, Latifa Al Husnain et al. "Biochar and jasmonic acid application attenuates antioxidative systems and improves growth, physiology, nutrient uptake and productivity of faba bean (Vicia faba L.) irrigated with saline water", Plant Physiology and Biochemistry, 2021
Crossref

8 words — < 1%

96 Nurunnaher Akter, Tanzina Afrin Brishty, M. Abdul Karim, M. Jalal Uddin Ahmed, M. Rafiqul Islam. "Leaf water status and biochemical adjustments as a mechanism of drought tolerance in two contrasting wheat (Triticum aestivum L.) varieties", Acta Physiologiae Plantarum, 2023
Crossref

8 words — < 1%

97 Rajesh Kumar Tewari, Praveen Kumar, Parma Nand Sharma, Sher Singh Bisht. "Modulation of oxidative stress responsive enzymes by excess cobalt", Plant Science, 2002
Crossref

8 words — < 1%

98 Zhixin Guo, Jingli Lv, Xiaoxing Dong, Nanshan Du, Fengzhi Piao. "Gamma-aminobutyric acid improves phenanthrene phytotoxicity tolerance in cucumber through the glutathione-dependent system of antioxidant defense", Ecotoxicology and Environmental Safety, 2021
Crossref
8 words — < 1%

99 dergipark.org.tr
Internet
8 words — < 1%

100 ijfaas.com
Internet
8 words — < 1%

101 iuss.org
Internet
8 words — < 1%

102 ndl.ethernet.edu.et
Internet
8 words — < 1%

103 prr.hec.gov.pk
Internet
8 words — < 1%

104 real-j.mtak.hu
Internet
8 words — < 1%

105 www.ispeco.org
Internet
8 words — < 1%

106 www.upr.edu.pk
Internet
8 words — < 1%

107 "Abiotic Stress Physiology of Horticultural Crops", Springer Nature, 2016
Crossref
7 words — < 1%

108 "Agronomic Crops", Springer Science and Business Media LLC, 2020
Crossref
7 words — < 1%

109 "New Frontiers in Stress Management for Durable Agriculture", Springer Science and Business Media LLC, 2020
Crossref
7 words — < 1%

110 "Rice Research for Quality Improvement: Genomics and Genetic Engineering", Springer Science and Business Media LLC, 2020
Crossref
7 words — < 1%

111 "Technological and Modern Irrigation Environment in Egypt", Springer Science and Business Media LLC, 2020
Crossref
7 words — < 1%

112 Cengiz Kaya, Levent Tuna, David Higgs. "Effect of Silicon on Plant Growth and Mineral Nutrition of Maize Grown Under Water-Stress Conditions", Journal of Plant Nutrition, 2006
Crossref
7 words — < 1%

113 Hossam S. El-Beltagi, Sikandar Shah, Sami Ullah, Sulaiman, Abdallah Tageldein Mansour, Tarek A. Shalaby. "Impacts of Ascorbic Acid and Alpha-Tocopherol on Chickpea (Cicerarietinum L.) Grown in Water Deficit Regimes for Sustainable Production", Sustainability, 2022
Crossref
7 words — < 1%

114 Jiaojiao Xu, Tao Liu, Feng Qu, Xiaoqing Jin, Na Huang, Jiaxuan Wang, Xiaohui Hu. "Nitric oxide mediates γ-aminobutyric acid-enhanced muskmelon tolerance to salinity–alkalinity stress conditions", Scientia Horticulturae, 2021
7 words — < 1%

Crossref

115 Jin Seok Yoon, Jae Yoon Kim, Dae Yeon Kim, Yong Weon Seo. "A novel wheat ASR gene, TaASR2D, enhances drought tolerance in Brachypodium distachyon", Plant Physiology and Biochemistry, 2020
Crossref
7 words — < 1%

116 Physiological Mechanisms and Adaptation Strategies in Plants Under Changing Environment, 2014.
Crossref
7 words — < 1%

117 Sami Ullah, Jehan Zada, Sajjad Ali. " Effect of Nephthyl Acetic Acid Foliar Spray on Amelioration of Drought Stress Tolerance in Maize (L.) ", Communications in Soil Science and Plant Analysis, 2016
Crossref
7 words — < 1%

118 Shamsul Hayat, Qaiser Hayat, Mohammed Nasser Alyemeni, Arif Shafi Wani, John Pichtel, Aqil Ahmad. "Role of proline under changing environments", Plant Signaling & Behavior, 2014
Crossref
7 words — < 1%

119 Yuxing Li, Boyang Liu, Yuxiao Peng, Chenlu Liu, Xiuzhi Zhang, Zhijun Zhang, Wei Liang, Fengwang Ma, Cuiying Li. "Exogenous GABA alleviates alkaline stress in Malus hupehensis by regulating the accumulation of organic acids", Scientia Horticulturae, 2019
Crossref
7 words — < 1%

120 atrium.lib.uoguelph.ca
Internet
7 words — < 1%

121 www.pakbs.org
Internet
7 words — < 1%

EXCLUDE QUOTES	ON	EXCLUDE SOURCES	OFF
EXCLUDE BIBLIOGRAPHY	ON	EXCLUDE MATCHES	< 7 WORDS
