# Supplementary material for: Physiology of gamma-aminobutyric acid treated Capsicum annuum L. (Sweet pepper) under induced drought stress
Source: PLoS One. 2023 Aug 17;18(8):e0289900. doi: 10.1371/journal.pone.0289900 (PMC10434925; doi:10.1371/journal.pone.0289900)
Supplement: Attachment — Submitted filename: Response to Reviewers.docx [file pone.0289900.s002.docx]

| **Serial#** | **Comments by reviewer#1** | **Response by author** |
| --- | --- | --- |
| 1 | The first 10 lines of the abstract should be concise. The results are not well written in the abstract. | The first 10 lines of the abstract have been concised. Results have been also been re-written in a proper manner. |
| 2 | Carefully check the entire text and ensure all scientific names are in italics. | All the text has been rechecked multiple times for mistakes and all the scientific names are now in italics. |
| 3 | The introduction is unnecessary is too long. Please significantly shorten it to 1.5 to 2 pages only. | The introduction is shortened to only 2 pages. |
| 4 | In the introduction, for drought and plant growth regulations, authors must add some most recent studies such as doi:10.1002/tpg2.20279; 10.1016/j.sajb.2023.05.018; 10.1080/15592324.2023.2186045; 10.1016/j.stress.2023.100152; etc. | Some of the most recent studies as mentioned by the respected reviewer have been added into the introduction. |
| 5 | The writing style is not consistent and must be checked and edited. | The writing style has been checked and edited multiple times and mistakes have been removed. |
| 6 | On what basis GABA dose was selected? | Based on the findings of the previous research studies GABA doses were applied in the present work. Moreover, prior to pot experiment a Petri dishes lab experiment was conducted, and the same GABA doses were applied. The applied doses proven effective in counteracting drought induced stress and increasing growth and physiological attributes. |
| 7 | Change Chloro to Chl throughout the MS. There are many repeated abbreviations. Please define all the abbreviations on the first mention in the main text, then use the abbreviated form. | Chloro is changed into Chl throughout the MS. Repeated abbreviations are removed and are now only defined on the first mention in the main text. |
| 8 | The presentation of results must be carefully improved. For example, authors can add %/folds numerical data for the increased/significant results. | Results which required improvement have been carefully improved and are now presented in figures. |
| 9 | So many figures and tables and some figures can be merged into 1 figure and arranged in different legends A/B/C/D etc. For instance, all graphs can be easily arranged in 2-3 figures. Please work on the figures and reduce the total number of figures. | Figures have been merged and arranged in legends as mentioned. |
| 10 | The discussion is too long and boring. Please shorten and provide mechanic insights behind the observed changes. | The discussion has been shortened and mechanic insight have been provided behind the observed changes. |
| 11 | Shorten the conclusion. | The conclusion have been improved and shortened. |
| 12 | The revised version should be carefully proofread. | Revised version has been carefully proofread. |

| **Serial#** | **Comments by reviewer#2** | **Response by author** |
| --- | --- | --- |
| 1 | the paper is very good the intro is very long - remove 50% of it | Thank you for such a great compliment respected sir. Introduction has been shortened and improved. |
| 2 | The extensive statistics have to move to supplm. (Regression .....) | Statistic have been moved to supplementary (Regression) |
| 3 | discussion need to condense and use updated references | Discussion has been condensed and updated references are also added. |
| 4 | similarity is high and need to reduce (attached file | Similarity has been reduced |

| **Serial#** | **Comments by reviewer#3** | **Response by author** |
| --- | --- | --- |
| 1 | Most part of the paper is like an easy, it must be written in scientific manner, better to use indirect form, and also the Use of “WE” is much more than the normal. | The paper has been re-written in scientific manner and indirect form. The use of “we” is removed. |
| 2 | The abstract contains most of the findings in parenthesis, remove these parentheses and make it a plane and easy to understand sentences. | Parenthesis in abstract are removed where possible and sentences are made easy to understand. |
| 3 | Add an introductory statement to the abstract and conclusive sentence at the end. Briefly discuss your methods too. | Introductory statement and conclusive sentence at the end has been added to the abstract and methods are discussed briefly. |
| 4 | Introduction and discussion has some irrelevant citations replace them with the following updated relevant works. | Irrelevant citations have been replaced with updated and relevant work. |
| 5 | An introduction of almost 2 pages would be sufficient to give an idea of the research done. Some data is given without providing proper citation. If this is not your findings add reference. | Introduction has been reduced to only 2 pages and proper citations are added to the data. |
| 6 | Methodology section is extensively elaborated. it is suggested to make it brief and concise. Each and every method should be cited, if it is not a first time invention. | Methodology section has been concised and each and every method that is not my work is also cited. |
| 7 | Try to merge all the tables in to a single table and add reference values. | The first 2 tables have been converted into graphs. |
| 8 | The graphs are properly prepared | Thank you respected sir. |
| 9 | The results are supported with the present day research which could be further improved with latest citations, it should be discuss thoroughly with existing review. | The results are further improved with latest citations and are thoroughly discussed with existing review. |

| **Serial#** | **Comments by editor** | **Response by author** |
| --- | --- | --- |
| 1 | Please ensure that your manuscript meets PLOS ONE's style requirements, including those for file naming. | The manuscript has been formatted according to the PLOS ONE style. |
| 2 | We suggest you thoroughly copyedit your manuscript for language usage, spelling, and grammar | I have thoroughly copyedited my manuscript and language, spelling and grammar mistakes have been removed. |
| 3 | Please ensure that you refer to Figure 1 in your text as, if accepted, production will need this reference to link the reader to the figure. | Figures have been updated and now referred to in the text. |
| 4 | We note you have included a table to which you do not refer in the text of your manuscript. Please ensure that you refer to Table 1 in your text; if accepted, production will need this reference to link the reader to the Table. | I have referred to tables in the correct format and are now in correct number. |
